# Extract of *Corallodiscus flabellata* attenuates renal fibrosis in SAMP8 mice via the Wnt/β-catenin/RAS signaling pathway

**DOI:** 10.1186/s12906-022-03535-y

**Published:** 2022-02-28

**Authors:** Bing Cao, Mengnan Zeng, Yanpo Si, Beibei Zhang, Yangyang Wang, Ruiqi Xu, Yanjie Huang, Weisheng Feng, Xiaoke Zheng

**Affiliations:** 1 Henan University of Chinese Medicine, 450046 Zhengzhou, China; 2 The Engineering and Technology Center for Chinese Medicine Development of Henan Province, 450046 Zhengzhou, China; 3 School of Pharmacy, Henan University of Chinese Medicine, 156 Jinshui East Road, 450046 Zhengzhou, China

**Keywords:** Aging, *Corallodiscus flabellata*, Kidney, Renal fibrosis, SAMP8, Senescence, Wnt/β-catenin/RAS

## Abstract

**Background:**

Fibrosis is one of the most common pathological features of the aging process of the kidney, and fibrosis in aging kidneys also aggravates the process of chronic kidney disease (CKD). *Corallodiscus flabellata* B. L. Burtt (*C. flabellata*, CF) is a commonly used botanical drug in Chinese folklore. However, few studies have reported its pharmacological effects. This study aimed to explore the effect of CF ethanol extract on renal fibrosis in SAMP8 mice and identify potentially active compounds.

**Methods:**

Senescence-accelerated mouse-prone 8 (SAMP8) were used as animal models, and different doses of CF were given by gavage for one month. To observe the degree of renal aging in mice using β-galactosidase staining. Masson staining and the expression levels of Col-I, α-SMA, and FN were used to evaluate the renal fibrosis in mice. The protein expression levels of Nrf2 pathway and Wnt/β-catenin/RAS pathway in the kidney were measured. And β-galactosidase (β-gal) induced NRK-52E cells as an in vitro model to screen the active components of CF.

**Results:**

The CF ethanol extract significantly inhibited the activity of renal β-galactosidase and the expression levels of Col-I, α-SMA, and FN in SAMP8 mice, and improved Masson staining in SAMP8 mice. CF remarkably reduced urinary protein, creatinine, urea nitrogen and serum levels of TNF-α and IL-1β in SAMP8 mice, and significantly increased the levels of SOD and GSH-Px. Moreover, CF activated the Nrf2 pathway and blocked the Wnt/β-catenin/RAS pathway in the kidneys of mice. Besides, 3,4-dihydroxyphenylethanol (SDC-0-14, 16) and (3,4-dihydroxyphenylethanol-8-O-[4-O-trans-caffeoyl-β-D-apiofuranosyl-(1→3)-β-D-glucopyranosyl (1→6)]-β-D-glucopyranoside (SDC-1-8) were isolated from CF, which reduced the senescence of NRK-52E cells, and maybe the active ingredients of CF playing the anti-aging role.

**Conclusions:**

Our experiments illuminated that CF ethanol extract may ameliorate renal fibrosis in SAMP8 mice via the Wnt/β-catenin/RAS pathway. And SDC-0-14,16 and SDC-1-8 may be the material basis for CF to exert anti-renal senescence-related effects.

**Supplementary Information:**

The online version contains supplementary material available at 10.1186/s12906-022-03535-y.

## Introduction

The global prevalence of chronic kidney disease (CKD) is increasing with the aging of the population and poses a significant burden to society [1–3]. The most common pathological manifestation of CKD is some form of renal fibrosis [4]. Likewise, renal fibrosis is also one of the signs of kidney aging [2]. Studies have shown that oxidative stress, inflammation, Wnt/β-catenin, renin angiotensin aldosterone system (RAS) and rapamycin (m-TOR) signaling are all related to CKD induced by senescence [5]. Renal fibrosis in aging kidneys is closely related to the activation of Wnt/β-catenin and RAS signaling pathway [6]. Upon activation of Wnts, β-catenin in the cytoplasm is translocated to the nucleus, where it binds to the lymphoid enhancer-binding factor (LEF)/the T-cell factor (TCF) transcription factor family and initiates the transcription of downstream target genes. Interestingly, the promoter region of the RAS gene also contains LEF/TCF binding sites, which allows β-catenin to promote the binding of LEF-1 to these sites [7]. Therefore, targeting the Wnt/β-catenin/RAS signaling pathway could be a potential therapeutic strategy for renal fibrosis [8, 9]. In recent years, the replacement therapy of renal fibrosis by natural products has attracted the attention of many scholars [10]. Xiaoyan Shen et al. found that ginsenoside Rg1 ameliorated glomerular fibrosis during kidney aging by inhibiting the activation of NLRP3 inflammasome in SAMP8 mice [11]. This study explored the effect of *C. flabellate* (CF) extract on renal fibrosis in SAMP8 mice from the Wnt/β-catenin/RAS pathway.

CF as a medicinal plant in China, was first recorded in the “Dian Nan Ben Cao.” The whole plant is commonly used for treating dysentery, premature ejaculation, seminal vesicle disease, and kidney disease in ethnic minority areas of China [12]. At present, there are few reports about CF in modern research. The pharmacological studies in our laboratory found that CF extract had diuretic effects and ameliorated lipopolysaccharide/D-galactosamine-induced liver failure and brain damage in rats [13, 14]. And phytochemical studies on it revealed that phenylethanoid glycosides and flavonoids were the main chemical components of CF [15, 16]. The aim of this study was to investigate the effect of CF extract on renal fibrosis in SAMP8 mice and to elucidate its possible mechanism, so as to provide experimental evidence for the treatment of kidney disease with CF described in ancient books.

## Materials and methods

### Collection and extraction of the plant material

“Yunnan Chinese Herbal Medicine” records that CF can be harvested throughout the year. The CF plants used in this experiment was harvested in Xixia County, Henan Province, China in September. It was identified by Professor Suiqing Chen from Henan University of Chinese Medicine, and the specimens were stored in the laboratory specimen library. The plants (1 kg) were refluxed with 50% ethanol (3 × 12 L, each 1 h), and the mixture was filtered with 16 layers of gauze. The combined filtrates were dried by rotary evaporation using a freeze drier. Finally, the percentage yield of 50% ethanol crude extract of CF was 15.5%. The dried extract was kept in a fridge until further use. The supplementary materials list the relevant data on the ingredient designation of CF extract.

Another separation process previously reported was used in this study to obtain the water elution fraction, 20% ethanol elution fraction, 30% ethanol elution fraction, and 40% ethanol elution fraction from CF [13]. The 40% ethanol fraction was separated using Sephadex LH-20 and silica gel column and purified by semi-preparative high-performance liquid chromatography (HPLC) to finally obtain compounds such as SDC-0-14,16, SDC-1-8, SDC-0-60 (p-hydroxybenzyl alcohol).

## Animals and administration

Six-month-old male SAMP8 and senescence-accelerated mouse resistant 1 (SAMR1) mice from the First Affiliated Hospital of Tianjin University of Traditional Chinese Medicine (Tianjin, China) were used in this study. The animals were housed under controlled light (12-h light/dark cycle), temperature (23-25 °C), and humidity (45-55%) conditions and received a standard diet and water ad libitum. A total of 48 SAMP8 mice were divided into four experimental groups (*n* = 12/group): SAMP8 model mice (M), low-dose CF ethanol extract-treated SAMP8 mice (CF-L, 387.5 mg/kg, intragastrically), medium-dose CF ethanol extract-treated SAMP8 mice (CF-M, 775 mg/kg, intragastrically), and high-dose CF ethanol extract-treated SAMP8 mice (CF-H, 1550 mg/kg, intragastrically). The mice in the SAMR1 control (Con, n = 10) and SAMP8 model groups (M) were treated with physiological saline (0.9%). All mice were treated orally for 1 month. All animal experiments were approved by the ethics committee of Henan University of Chinese Medicine and performed under the institutional guidelines (Henan, China Approval Number: HACTCM-2018009060-19). During the experiment, there was one mouse death in each of the Con and M group, and two mice died in the CF-H group.

### Sample collection

At the end of the experiment, the mice were housed individually in metabolic cages for 12-h urinary collection. The mice were then anesthetized with isoflurane, and blood samples were collected through retro-orbital bleeding. The kidneys from each mouse were then surgically removed and kept at -80 °C until the analyses.

### Cell culture and *in vitro* study

NRK-52E cells purchased from the Cell Bank of the Chinese Academy of Sciences (Shanghai, China) were cultured to investigate the effect of CF extract on cell aging caused by D-galactose (D-gal, S11050, Yuanye, Shanghai, China). The NRK-52E cells were grown in Dulbecco’s modification of Eagle’s medium Dulbecco (DMEM, 12,100,046, Thermo Fisher, Massachusetts, USA) supplied with 10% fetal bovine serum in an incubator at 37 °C and in the presence of 5% CO_2_. Cells were grown in 96-well plates or 6-well plates to 80–85% confluence and then treated with growth media containing different drug combinations: Media + D-gal (20 mg/mL), Media + D-gal + CF (10, 25, 50, 100 µg/mL) or Media + D-gal + monomeric compound (10 µM) respectively for 48 h. Subsequently, the methyl thiazolyl tetrazolium (MTT) assay was used to detect cell viability, and β-galactosidase staining was used to observe cell senescence.

### Senescence-associated β-galactosidase staining

Frozen kidneys from mice sliced into 10-µm-thick sections and NRK-52E cells were stained with senescence-associated β-galactosidase (SA-β-gal, C0602, Beyotime Biotechnology, Shanghai, China) following the manufacturer’s protocols.

### Histological analysis

The kidney sections were fixed with 4% buffered paraformaldehyde, and 10-µm-thick paraffin-embedded sections were stained with Masson’s trichrome (G1006, Servicebio, Wuhan, China) and observed microscopically. The blue-colored areas in Masson’s trichrome–stained sections were measured quantitatively from six randomly selected fields and analyzed by Image-Pro Plus 6.0 software.

### Biochemical measurements

The serum and kidney homogenate samples were thawed to room temperature, and the levels of superoxide dismutase (SOD, CSB-E08556m, Wuhan Huamei, Wuhan, China), glutathione peroxidase (GSH-Px, A005-1-2, Nanjing Jiancheng, Nanjing, China), interleukin-1β (IL-1β, RK00006, ABclonal, Wuhan, China), tumor necrosis factor-α (TNF-α, RK00027, ABclonal, Wuhan, China), urea nitrogen (C013-2-1, Nanjing Jiancheng, Nanjing, China), creatinine (Cr, C011-2-1, Nanjing Jiancheng, Nanjing, China) in mouse serum, total urinary protein (C035-2-1, Nanjing Jiancheng, Nanjing, China) in mouse urine and the expression levels of collagen type I (Col-I, MU30364, Bio-Swamp, Wuhan, China), α-smooth muscle actin (α-SMA, MU30359, Bio-Swamp, Wuhan, China), fibronectin (FN, MU30179, Bio-Swamp, Wuhan, China) in mouse kidney tissues were measured sequentially according to the protocol of the assay kit manufacturer.

### Immunohistochemical analysis

The immunohistochemical analysis was performed using the routine method [17]. The antibodies used included the following: Wnt4 (14371-1-AP, Proteintech, Chicago, USA), β-catenin (17565-1-AP, Proteintech, Chicago, USA), type 1 angiotensin II receptors (AGTR1, 25343-1-AP, Proteintech, Chicago, USA), p-nuclear factor erythroid 2–related factor 2 (*p*-Nrf2, ab76026, Abcam, Cambridge, UK), *p*-c-Fos (ab27793, Abcam, Cambridge, UK), connective tissue growth factor (CTGF, GB11078, Servicebio, Wuhan, China). The sections were observed under a microscope (Olympus, Tokyo, Japan). Measure the area and integrated optical density (IOD) of the area with tan expression using Image-Pro Plus 6.0 software, and calculate the mean optical density (MOD, MOD = IOD/area) for semi-quantitative analysis.

### Western blot analysis

Western blot assay was conducted as described in a previous study [18]. Briefly, the kidney tissues were homogenized in lysis buffer and quantified using a Bradford Protein Assay Kit (AR0197, Boster Biological Technology, Wuhan, China). The homogenates were then subjected to sodium dodecyl sulfate-polyacrylamide gel electrophoresis, transferred to polyvinylidene fluoride membrane, and blocked in blocking buffer (4% nonfat dry milk) for 90 min. They were then incubated with primary antibodies (Wnt4; renin; AGTR1; *p*-Nrf2; *p*-c-Fos; Kelch like Ech associated protein 1 (Keap1, GB11847, Servicebio, Wuhan, China); β-actin (AC026, Abclonal, Wuhan, China); and glyceraldehyde-3-phosphate dehydrogenase (GAPDH, AC033, Abclonal, Wuhan, China)) overnight at 4℃, followed by incubation with an appropriate fluorescence-conjugated secondary antibody for 1 h at room temperature. The proteins of interest were scanned with an Odyssey IR scanner (LI-COR Biosciences, Nebraska, USA), and the signal intensities were quantified using Image Studio software. The protein levels were normalized against the β-actin or GAPDH.

### UPLC-Q-TOF-MS analysis for kidney samples

The renal tissues were weighed and homogenized in ice-cold physiological saline (w/v = 1:1). Then, 1 mL of acetonitrile was added to 200 µL of tissue homogenate samples, followed by ultrasonic extraction for 30 min. The extract was centrifuged at 12,000* g* and 4℃ for 10 min. The supernatant was taken into the vial for analysis. Chromatographic separation was carried out on ultraperformance liquid chromatography (UPLC) (Dionex UltiMate 3000 System, Thermo Scientific, Massachusetts, USA), with the LC system comprising an Acclaim RSLC 120 C_18_ column (2.2 μm, 2.1 × 100 mm; Thermo Scientific). The mobile phase consisted of solvent A (acetonitrile) and water with 0.1% formic acid (B). The separation was performed by gradient elution as follows: 10-70% A from 0 to 3 min, 70-78% A from 4 to 13 min, 78-90% A from 14 to 15 min, 90%-10% A from 15 to 16 min, and 10% A from 16 to 20 min. The injection volume of the test sample was 2 µL. The mass spectrometry (MS) analysis was performed by using an ESI source under the following conditions: the capillary voltage in the positive mode was 3.5 kV, and the capillary voltage in the negative mode was 3.2 kV. The pressure of the nebulizer was 2.0 bar, the temperature of the dry gas was 230 ℃, and the flow rate was 8 L/min.

### Statistical analysis

The acquired raw data from ultra-performance liquid chromatography coupled to quadrupole time-of-flight mass spectrometry (UPLC-Q/TOF-MS) analysis were first preprocessed using profile analysis (version 2.1, Bruker, Germany). The “bucket table” was obtained and imported into the SIMCA-P software (version13.0 Umetrics AB, Sweden) for principal component analysis (PCA). Other results were presented as mean ± standard deviation (SD). The data were processed using IBM SPSS Stastic 26.0. The normal distribution of the data used the Levene test, which required the average value, *P* > 0.05; the one-way analysis of variance was used for comparison between groups. A *P* value less than 0.05 was considered statistically significant.

## Results

### CF improved the aging and fibrosis of the kidneys in SAMP8 mice

**Fig. 1:**
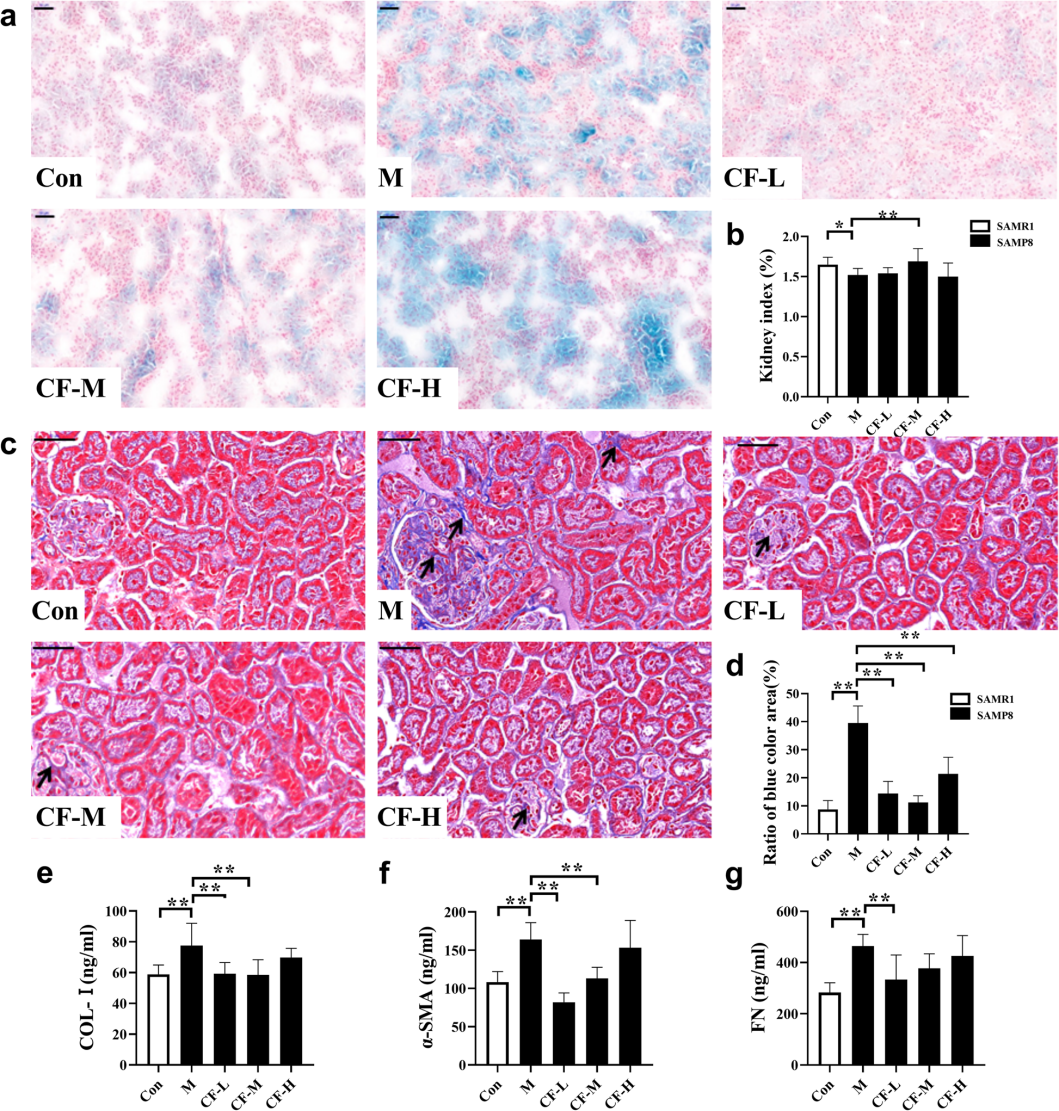
CF improved the aging and fibrosis of the kidneys in SAMP8 mice. **a** The activity of SA-β-gal in frozen sections of kidneys. Scale bars: 50 μm, magnification 200×. **b** Changes in the mouse kidney index (kidney index = kidney weight/mouse weight × 100%). **c** Masson’s trichrome staining of kidneys. Renal fibrosis was expressed by collagen deposition (blue color area) in Masson’s trichrome staining. Scale bars: 50 μm, magnification 400×. **d** Semi-quantification of renal interstitial fibrosis. The ratio of the blue color area to the field area from Masson’s trichrome-stained sections. **e-g** The expression levels of Col-I, α-SMA, and FN in kidney tissue were detected by ELISA. Con: SAMR1 mice; M: SAMP8 mice; CF-L: Treated with low-dose CF ethanol extract; CF-M: Treated with medium-dose CF ethanol extract; CF-H: Treated with high-dose CF ethanol extract; Values expressed as mean ± SD; ^*^P < 0.05 and ^**^P < 0.01 vs. M group (*n* = 6)

To investigate the effect of CF on the kidneys of SAMP8 mice, SA-β-gal activity was measured in the kidneys. In Fig. 1a, The SA-β-gal activity in kidney tissues significantly increased in the M group (blue enhancement). Administration of CF-L and CF-M significantly inhibited β-galactosidase activity in the kidneys of SAMP8 mice while the improvement by CF-H was not significant. Also, the kidney index of the model group was significantly lower than that of the control group (Fig. 1b, *P* < 0.05). After treatment with CF-M, the kidney index of SAMP8 mice were improved (*P* < 0.01). Next, the results of Masson’s trichrome staining (Fig. 1c, *P* < 0.01) and the expression of fibrosis indicators Col-I, α-SMA, and FN revealed that M-group mice had more severe renal fibrosis than Con-group mice, and CF-L and CF-M significantly attenuated renal fibrosis in SAMP8 mice (Fig. 1e-g, *P* < 0.01).

### CF improved renal function, oxidative stress and inflammation levels in SAMP8 mice

**Fig. 2:**
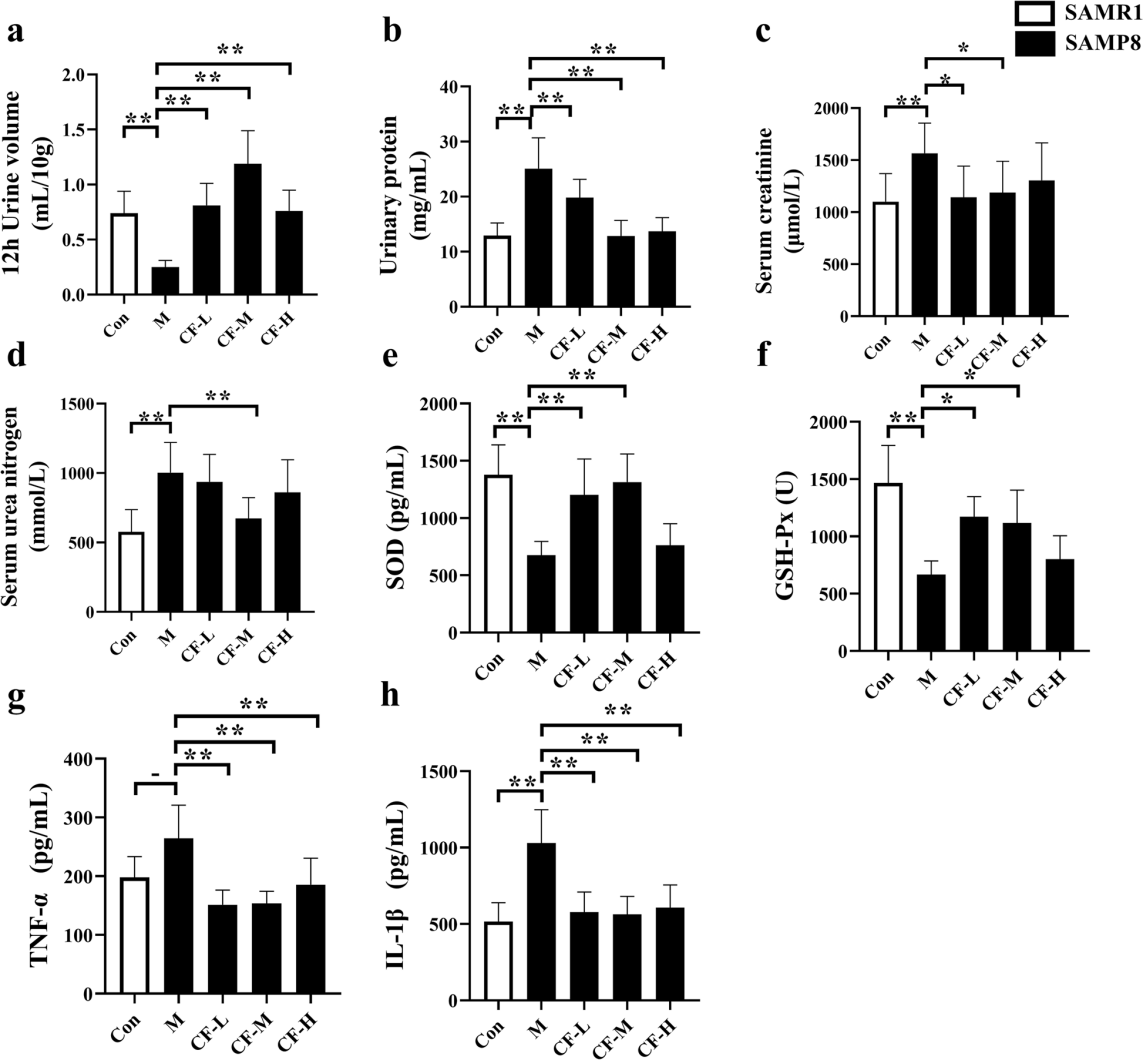
CF improved renal function, oxidative stress and inflammation levels in SAMP8 mice. **a-d** Effect of CF on the urine volume, urine protein levels, and serum Cr and urea nitrogen levels, respectively. **e-h** The level of SOD, GSH-Px, TNF-α and IL-1β in serum, respectively. Con: SAMR1 mice; M: SAMP8 mice; CF-L: Treated with low-dose CF ethanol extract; CF-M: Treated with medium-dose CF ethanol extract; CF-H: Treated with high-dose CF ethanol extract; Values expressed as mean ± SD; ^*^P < 0.05 and ^**^P < 0.01 vs. M group (*n* = 5–12)

Besides, the renal function indicators of each group of mice were tested, which included urine volume in mice for 12 h, Cr and urea nitrogen levels in the serum, and urine protein levels. As shown in Fig. 2a-d, the urine protein, serum Cr and serum urea nitrogen levels of the model group were significantly higher than those of the control group (Fig. 2a, *P* < 0.01), while the urine volume of the model group was significantly lower than that of the control group (Fig. 2b-d, *P* < 0.01). CF supplementation down-regulated the levels of urine protein, serum Cr and urea nitrogen in the model group (Fig. 2b-d, *P* < 0.05 or *P* < 0.01) while increasing the urine output of model mice. Collectively, the results indicated that SAMP8 mice had kidney damage associated with aging, and treatment with CF effectively ameliorated this injury. Next, whether CF beneficially modulated oxidative stress and inflammation in the kidneys of rapidly aging mice was explored. As shown in Fig. 2e-h, the levels of SOD and GSH-Px in the kidneys of the M group were significantly lower than those of the Con group, and the levels of IL-1β were significantly higher than those of the Con group. After CF treatment, the above indicators have been improved, especially the improvement effect of CF-L and CF-M is better.

### CF activated the Nrf2 pathway in the kidney of SAMP8 mice

**Fig. 3:**
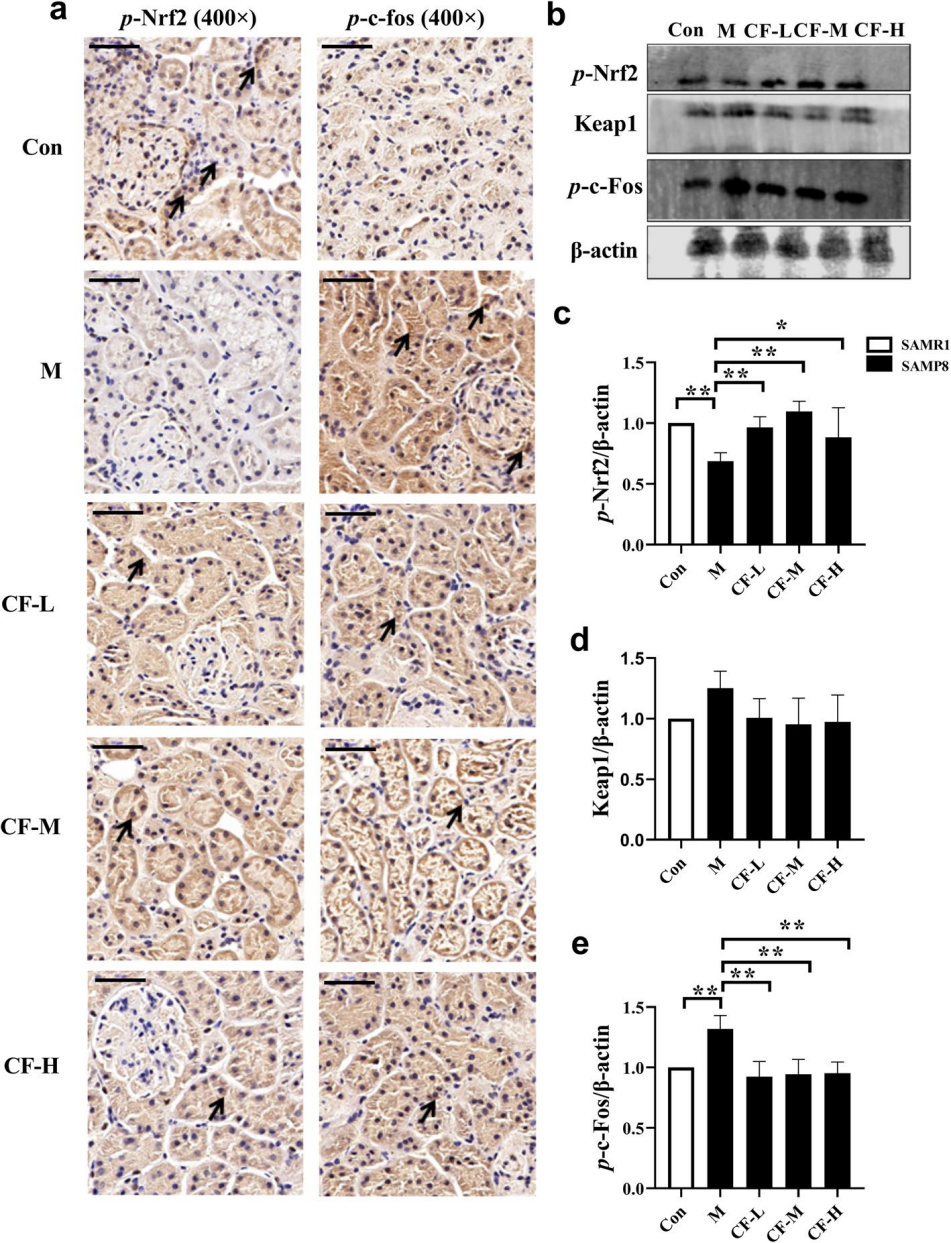
CF activated the Nrf2 pathway in the kidney of SAMP8 mice. Scale bars: 50 μm. **a** The localization of *p*-Nrf2, *p*-c-Fos in the kidney was detected by immunohistochemistry. **b-e** Protein strip and quantification of *p*-Nrf2, Keap1, and *p*-c-Fos in the kidney were detected by western blotting. Arrows indicate positive expression. Con: SAMR1 mice; M: SAMP8 mice; CF-L: Treated with low-dose CF ethanol extract; CF-M: Treated with medium-dose CF ethanol extract; CF-H: Treated with high-dose CF ethanol extract; Data represent the mean values ± SD from three independent experiments. ^*^P < 0.05 and ^**^P < 0.01 vs. M group (*n* = 3 or *n* = 6)

The expression level of *p*-Nrf2 was measured in kidney tissues to investigate whether the Nrf2 pathway was involved in the protective effect of CF (Fig. 3a-c) in the present study. The expression level of *p*-Nrf2 significantly decreased in the SAMP8 group alone (*P* < 0.01, Fig. 3c), and the downregulation was reversed to some extent by the treatment of CF crude extract. There was no significant change in the expression level of Keap1 among the groups (Fig. 3b and d). Further, the expression level of *p*-c-Fos was detected and analyzed by immunohistochemical and Western blot analyses. It was found that the expression level of *p*-c-Fos in the kidneys of mice in the M group was significantly higher than that in the Con group; while CF treatment significantly reduced the expression levels of *p*-c-Fos in the kidneys of the mice (Fig. 3a-b and e).

### CF attenuated Wnt/β-catenin/RAS signaling activation in the kidney of SAMP8 mice

**Fig. 4:**
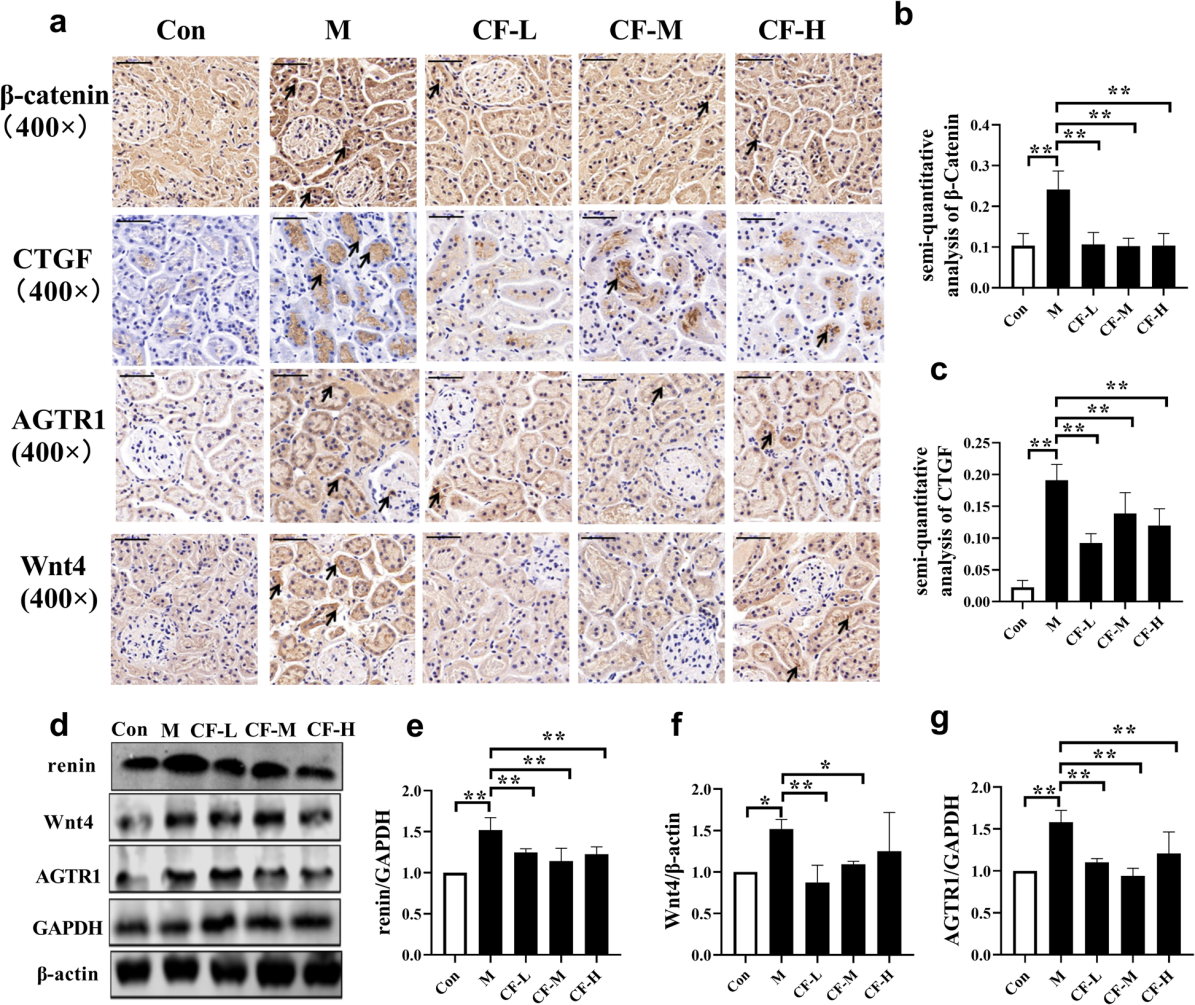
CF attenuated Wnt/β-catenin/RAS signaling activation in the kidney of SAMP8 mice. Scale bars: 50 μm. The localization of AGTR1, Wnt4, β-catenin, and CTGF in the kidney was detected by immunohistochemistry (**a**), and β-catenin (**b**) and CTGF (**c**) were semi-quantitatively analyzed. Protein strip (**d**) and quantification of renin (**e**), Wnt4 (**f**), and AGTR1 (**g**) in the kidney were detected by western blotting. Con: SAMR1 mice; M: SAMP8 mice; CF-L: Treated with low-dose CF ethanol extract; CF-M: Treated with medium-dose CF ethanol extract; CF-H: Treated with high-dose CF ethanol extract; Arrows indicate positive expression. Data represent the mean values ± SD. ^*^P < 0.05 and ^**^P < 0.01 vs. the M group (*n* = 3)

In order to further explored the potential mechanism of CF exerting anti-fibrosis effect. The localization of protein was performed using immunohistochemistry. As shown in Fig. 4a, the expression level of Wnt4 and β-catenin was induced predominantly in renal tubular cells. Similar results were observed when AGTR1, the downstream pathway targets of Wnt signaling, were assessed (Fig. 4a). After that, the protein expression level was quantified, and the results showed that Wnt4, β-catenin, renin, and AGTR1 proteins were accumulated in the kidney tissue of mice in the model group, whereas CF significantly inhibited those alterations (Fig. 4b-f ). Furthermore, the expression level of CTGF was also located and quantitatively analyzed. Consistent with the aforementioned results, CTGF was mainly expressed in the renal tubules, and CF markedly inhibited the activity of CTGF (Fig. 4d g).

### CF and its compounds inhibited the senescence of NRK-52E cells induced by D-gal

**Fig. 5:**
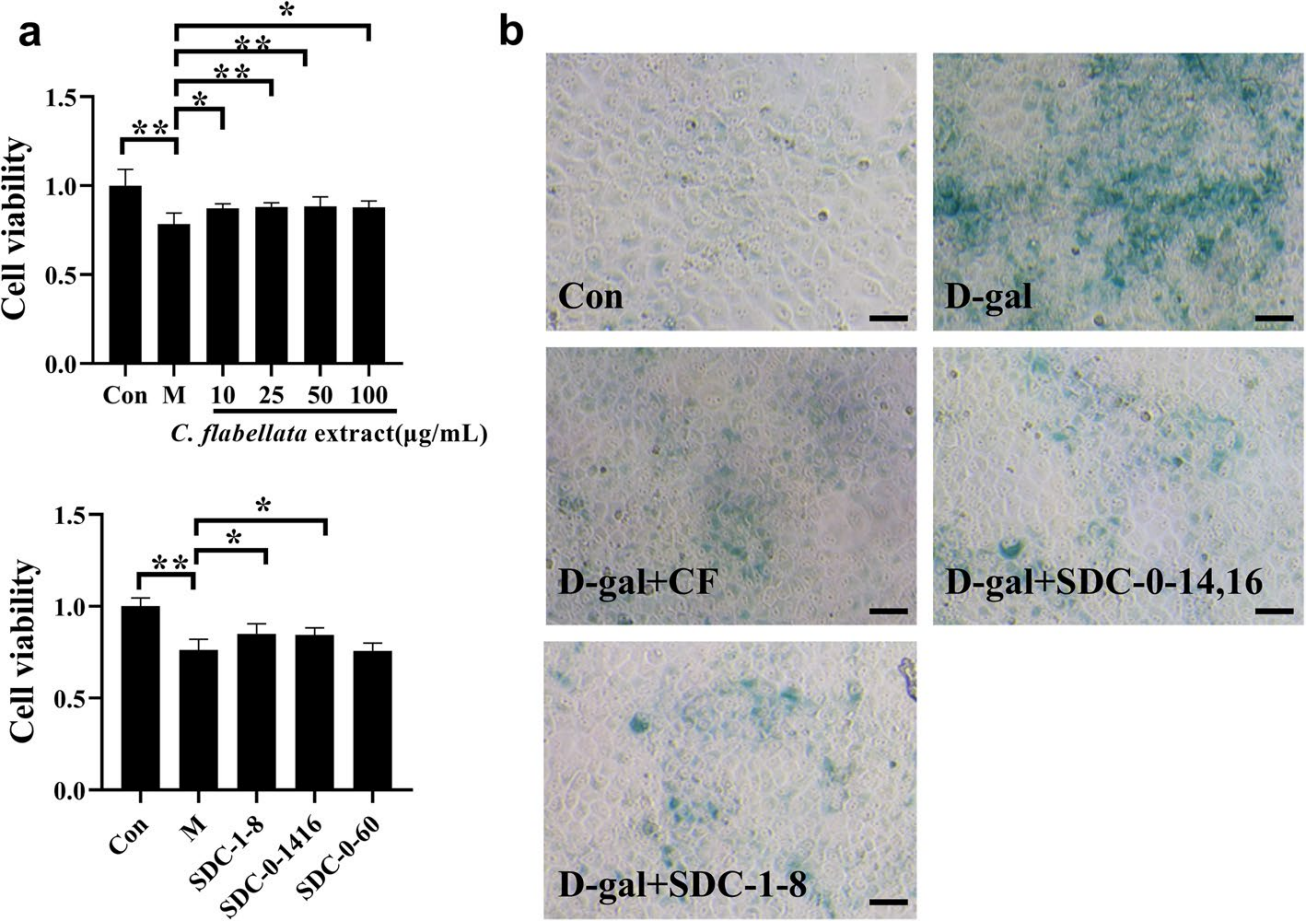
CF and its compounds inhibited the senescence of NRK-52E cells induced by D-gal. **a** Detection of cell viability of CF and its chemical components. **b** β-galactosidase staining of NRK-52E cells. Scale bars: 50 μm. CF: *C. flabellate* extract; D-gal: D-galactose; SDC-0-14,16: 3,4-dihydroxyphenylethanol; SDC-1-8: (3,4-dihydroxyphenylethanol-8-O-[4-O-trans-caffeoyl-β-D-apiofuranosyl-(1→3)-β-D-glucopyranosyl (1→6)]-β-D-glucopyranoside; SDC-0-60: p-hydroxybenzyl alcohol. Data represent the mean values ± SD from three independent experiments. ^*^P < 0.05 and ^**^P < 0.01 vs. the D-gal group

D-gal-induced NRK-52E cells were used to screen certain active ingredients in CF, to determine the material basis for the treatment of senile kidney by the extract of CF. The results showed that the CF enhanced cell viability in a dose-dependent manner, and the compounds SDC-0-14,16 and SDC-1-8 isolated from CF had better effects on cell proliferation. Besides, 25 µg/mL CF and the compounds SDC-0-14,16 and SDC-1-8 all displayed the effect of inhibiting β-galactosidase activity (Fig. 5).

### CF improved PCA analysis of kidney tissue in SAMP8 mice

**Fig. 6:**
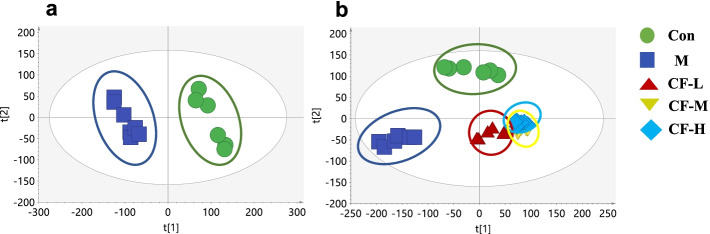
PCA score plot of kidney homogenate extract in positive modes. **a** PCA score plot for the Control and M groups (R2X = 0.542, Q2 = 0.38); **b** PCA score plot for all groups (R2X = 0.63, Q2 = 0.489). Con: SAMR1 mice; M: SAMP8 mice; CF-L: Treated with low-dose CF ethanol extract; CF-M: Treated with medium-dose CF ethanol extract; CF-H: Treated with high-dose CF ethanol extract;

Principal components analysis (PCA) was performed to explore the effects of CF on SAMP8 mice. As shown in Fig. 6a, there was an obvious grouping between the control and model groups (R2X = 0.542; Q2 = 0.38), suggesting that the endogenous metabolites of SAMP8 mice were different from SAMR1 mice, which causes them to deviate from the SAMR1 group. As shown in Fig. 6b, the different CF groups also clustered into different classes from the control and model groups, and the CF groups moved closer to the control group, indicating that the endogenous metabolites of the CF-intervened SAMP8 mice were similar to those of the SAMR1 mice.

## Discussion

Aging plays an important role in the progression of CKD [19]. As CKD fibrosis progresses, senescent cells express and secrete pro-fibrotic factors (TGF-β, CTGF, and so forth) and pro-inflammatory factors (IL-1β, IL-6, TNF-α, and so forth), which are senescence-associated secretory phenotype factors, thereby accelerating renal fibrosis [20, 21]. At present, traditional Chinese medicine has achieved good results in treating renal fibrosis with fewer side effects [22]. This study explored the interventional effects of CF extract on renal fibrosis in SAMP8 mice.

The mouse strain selected for this study was SAMP8, which was developed based on the lifespan, senescence, and pathological phenotypic grading scores of AKR/J mice and was an accelerated aging model solely of genetic origin [23, 24]. Studies have shown that the changes in the renal pathology of SAMP8 mice (9 months) include tubulointerstitial fibrosis and focal segmental glomerulosclerosis [25]. And it was found that renal fibrosis in SAMP8 mice was age-related [6]. Therefore, we tested the activity of β-galactosidase and the level of renal fibrosis in SAMP8 mice, and found that CF-L and CF-M improved kidney fibrosis and β-galactosidase activity in SAMP8 mice (Fig. 1). And we also tested the urine output of the mice. Consistent with previously published studies indicating that CF obtained by two different processes had diuretic effects [13], the extract significantly increased the urine volume of SAMP8 mice. Also, the present study showed that supplementation with CF improved the renal function, the activity of antioxidant enzymes, and levels of inflammatory factors in SAMP8 mice (Fig. 2).

The Keap1–Nrf2 system gained the attention of many scholars in recent years due to its antioxidant and anti-inflammatory properties. Its pharmacological potential in treating kidney diseases had been extensively studied in nonclinical and clinical studies [26]. Nrf2 is a master transcriptional regulator for genes related to redox status and antioxidant effects [27]. Studies have shown that phosphorylation is required for Nrf2 activation and target gene induction [28]. Activating Nrf2 improved CKD progression by preventing oxidative stress and maintaining cellular redox homeostasis [29]. As a key transcription factor, Nrf2 plays a crucial role in defense against oxidative stress by regulating its downstream antioxidants and detoxification enzymes [30]. Kim et al. reported that resveratrol, as a potent Nrf2 activator, ameliorated aging-related progressive renal injury [31]. In the present study, CF improved the attenuation of Nrf2 in the kidney of SAMP8 mice without affecting the expression level of Keap1, suggesting that CF crude extract might improve oxidative damage in the kidneys by activating the Nrf2 pathway (Fig. 3).

Renal fibrosis is characterized by excessive extracellular matrix (ECM) deposition leading to the formation of scars in the renal parenchyma [32]. Transforming growth factor-β (TGF-β) is thought to be a key cytokine in fibroblast overactivation [33, 34]. Of course, targeting only the TGF-β signaling pathway is insufficient to reduce renal fibrosis. Some studies indicated that CTGF, Wnt/β-catenin, renin-angiotensin system, oxidative stress, and so forth, were implicated in renal fibrosis [35–37]. Wnt/β-catenin is an evolutionarily conserved signaling pathway involved in the regulation of tissue homeostasis, organ development, and injury repair [38]. Cisternas et al. discussed the pro-fibrotic effect of Wnt signaling in both skeletal muscle and kidney [39]. Mounting evidence established that the Wnt/β-catenin signaling pathway plays a crucial role in regulating the development and progression of renal fibrotic lesions following injury [40–42]. The Wnt/β-catenin signal is relatively silent in the kidneys of healthy adults and is activated once the kidneys are subjected to various kinds of damages [43]. In mammals, the Wnt family has at least 19 family members critical for kidney development. And at least 15 of these family members are differentially upregulated in the aging kidney, including Wnt4 [6]. The results of the present study showed that the expression level of Wnt4 protein in the kidneys of SAMP8 mice was significantly higher than that in SAMR1 mice, which was verified by both Western blot and immunohistochemical analyses (Fig. 4a, d and f). Coincidentally, the expression level of β-catenin protein was also upregulated. Also, both Wnt4 and β-catenin were expressed in renal tubular epithelial cells, as revealed by the immunohistochemical analysis (Fig. 4a and f). Wnt/β-catenin elicited renal fibrosis by inducing multiple fibrogenic genes such as RAS components, matrix metalloproteinase-7 (MMP-7), plasminogen activator inhibitor 1 (PAI-1), and Snail1 [37]. Zhou et al. described Wnt/β-catenin as the major upstream regulator, which controls the expression of all tested RAS components in the kidneys [44]. Zhou et al. used a 5/6 nephrectomy (5/6 NX) rat model to show that the expression levels of major components of RAS in the brain and kidneys, such as angiotensinogen, angiotensin-converting enzyme, and angiotensin II AT1-receptor, was significantly upregulated. The upregulated expression level was inhibited by a central blocker of Wnt, which was an adeno-associated virus vector overexpressing the *DKK1* gene [45]. Similarly, the present study found that AGTR1 was still expressed in the renal tubular epithelium, and the expression levels of renin and AGTR1 in the kidney tissues of SAMP8 mice were significantly greater than those in SAMR1 mice (Fig. 4a and d g). These results indicated that the Wnt/β-catenin/RAS signaling pathway was activated in the kidneys of SAMP8 mice, and the CF effectively inhibited the activation of this pathway.

CTGF exerts multiple biological functions, including promoting mitosis of chemotactic cells, inducing adhesion, and promoting cell proliferation and ECM synthesis [35, 46]. CCN2 (CTGF) modulated Wnt signaling by binding to low-density lipoprotein receptor-related protein 5/6 (LRP5/6) to further mediate fibrosis [39, 47]. Other studies used gene silencing to discover and confirm that Nrf2 regulated the Wnt pathway by regulating the expression level of CTGF, affecting renal interstitial disease. Also, Nrf2 regulated the CTGF transcription level mainly via CTGF transcriptional regulator c-Fos [48]. The immunohistochemical analysis revealed that CTGF and *p*-c-Fos were expressed mainly in renal tubular epithelial cells, and *p*-c-Fos was also distributed in glomeruli. The quantitative analysis showed that the expression levels of both proteins was inhibited by CF (Figs. 3b and e and 4a and c). Finally, the PCA analysis was used to further verify that CF improved kidney damage in SAMP8 mice (Fig. 6). The present study provided evidence that the CF not only activated Nrf2 signaling but also relied on Nrf2 to balance oxidative stress and inflammation, inhibited Wnt/β-catenin/RAS signaling, and improved kidney aging and renal fibrogenesis. However, we also found inconsistent improvement in the effect of CF on Masson and SA-β-gal staining in this experiment. It is speculated that this may be due to the inconsistent progression of renal aging and renal fibrosis in SAMP8 mice. The degree of renal aging in the mice in this experiment may be more serious than fibrosis. Therefore, the ameliorating effect of CF-H on renal aging in SAMP8 mice was not as obvious as that of renal fibrosis.

As a reducing monosaccharide, D-galactose is widely used in various age-related diseases *in vivo* and *in vitro*. D-gal was found to cause senescence and injury in NRK-52E cells [49], induce senescence of human kidney proximal tubular epithelial cells (HKC-8 cells), and increase the expression levels of two renal fibrosis marker proteins FN and α-SMA [6]. In this study, D-gal was used to induce NRK-52E cells *in vitro*. Using MTT assay and β-galactosidase staining to detect the compounds isolated from CF, it was found that SDC-0-14,16 and SDC-1-8 enhanced the cell viability induced by D-gal and inhibited D-gal induced cell senescence (Fig. 5). These suggested that SDC-0-14,16 and SDC-1-8 may be the material basis for CF to delay kidney aging. SDC-1-8 is one of the phenylethanoid glycosides. phenylethanoid glycosides were found to extend the life span of Caenorhabditis Elegans [50], with anti-aging, [51] and neuroprotective effects [52–54]. These provide a direction for our follow-up study on the pharmacological effects of CF.

## Conclusions

In conclusion, *in vivo* studies showed that CF reduced renal fibrosis in elderly mice. Some potential active ingredients were found in *in vitro* experiments. These findings provided pharmacological support for treating kidney disease using CF and a direction for further research on the active ingredients of CF.

## Supplementary Information

**Additional file 1.**

**Additional file 2.**

**Additional file 3.**

## Data Availability

All data generated or analyzed during this study are included in this published article. Raw data of this study can be asked to the corresponding author if needed.

## Abbreviations

CKD: Chronic kidney disease
*C. flabellate*: *Corallodiscus flabellata* B. L. Burtt
RAS: Renin-angiotensin system
SDC-0-14, 16: 3,4-dihydroxyphenylethanol
SDC-1-8: (3,4-dihydroxyphenylethanol-8-O-[4-O-trans-caffeoyl-β-D-apiofuranosyl-(1→3)-β-D-glucopyranosyl (1→6)]-β-D-glucopyranoside
SDC-0-60: p-hydroxybenzyl alcohol
HPLC: High-performance liquid chromatography
NRK-52E cells: normal rat kidney epithelial cells
DMEM: Dulbecco’s modification of Eagle’s medium Dulbecco
D-gal: D-galactose
SAMP8: Senescence-accelerated mouse-prone 8
SAMR1: Senescence-resistant mouse-prone 1
SOD: superoxide dismutase
IL: interleukin
TNF-α: tumor necrosis factor-α
Col-I: Collagen type I
α-SMA: α-smooth muscle actin
FN: fibronectin
Cr: creatinine
GSH-Px: glutathione peroxidase
AGTR1: type 1 angiotensin II receptors
CTGF: connective tissue growth factor
p-Nrf2: p-nuclear factor erythroid 2–related factor 2
Keap1: Kelch-like Ech-associated protein-1
GAPDH: glyceraldehyde-3-phosphate dehydrogenase
UPLC-Q/TOF-MS: ultra-performance liquid chromatography coupled to quadrupole time-of-flight mass spectrometry
PCA: principal component analysis
SD: standard deviation
SA-β-gal: senescence-associated β-galactosidase
ECM: excessive extracellular matrix
TGF-β: transforming growth factor-β
HKC-8 cells: Human kidney proximal tubular epithelial cells
