# Supplementary material for: Extract of Corallodiscus flabellata attenuates renal fibrosis in SAMP8 mice via the Wnt/β-catenin/RAS signaling pathway
Source: BMC Complement Med Ther. 2022 Feb 28;22:52. doi: 10.1186/s12906-022-03535-y (PMC8887028; doi:10.1186/s12906-022-03535-y)
Supplement: Supplementary file 1 — Additional file 1. [file 12906_2022_3535_MOESM1_ESM.docx]

**Supplementary Materials**

***High-Performance Liquid Chromatography-*** ***Electrospray Mass Spectrometry（HPLC-*** ***ESI-MS）***

The 50% EtOH crude extract of CF was measured by high-performance liquid chromatography coupled with electrospray mass spectrometry (HPLC/ESI-MS). The chromatographic column is an Acclaim TM RSLC 120 C_18_ column (2.2 µm, 2.1 × 100 mm; Thermo Scientific, Waltham, MA, USA). The mobile phase was composed of acetonitrile (A) and 0.1% formic acid–water (B). Then set the gradient elution procedure (0-1 min, 92-85% B; 1-8 min, 85-80% B; 8-20 min, 80-75% B; 20-28 min, 75-70% B; 28-30 min, 70-60% B). The flow rate of the mobile phase was 0.3 mL/min. The column temperature was maintained at 40℃, and the sample manager temperature was set at 4℃. The sample size is 2 μL. Mass spectrometry was performed on a Quadrupole Time-of-Flight Mass Spectrometer (Q-TOF-MS; maXis HD, Bruker, Karlsruhe, Germany) using an ESI source. The scanning mass range was from 50 to 1500 (m/z) with a spectra rate of 1.00 Hz. The capillary voltage was set at 3500 V and 3200 V for positive and negative modes. The pressure of the nebulizer was set at 2.0 Bar, the dry gas temperature at 230℃, and the continuous dry gas flow rate at 8 L/min. The acquired HPLC/ESI-MS chromatogram were compared with standard or compounds separated from CF in the laboratory with retention time or quasi-molecular ion peaks to determine the compounds contained in CF extract. The unspecified components in the HPLC/ESI-MS chromatogram are unknown.

**Fig. S1**. HPLC/ESI–MS chromatogram of CF extract in positive mode.


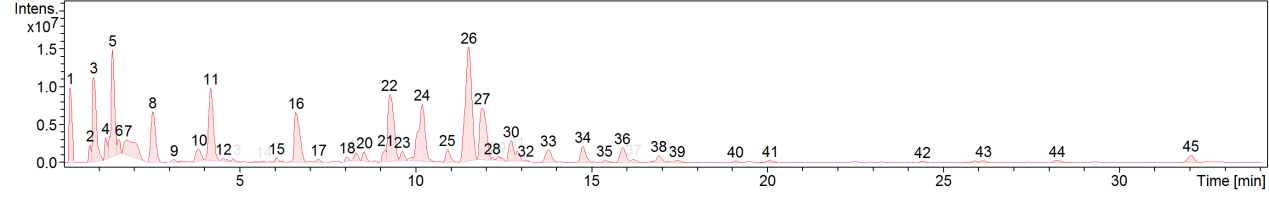


**Table1．**Ingredients identification table of CF extract

| No. | t_R_(min) | Formula | Compounds | |
| --- | --- | --- | --- | --- |
| 4 | 1.2 | C_25_H_38_O_17_ | 1'-O-β-D-(3,4-dihydroxyphenylethyl)-β-D-celyose(1→3')-β-D-glucose-(1→6')-glucoside | |
| 5 | 1.4 | C_14_H_20_O_8_ | 3,4-dihydroxyphenylethanol-8-O-β-D-glucoside | |
| 9 | 3.1 | C_19_H_28_O_11_ | 1′-O-β-D-(3,4-dihydroxyphenethyl)-β-D-apigenose- (1 → 2 ′)-glucoside | |
| 10 | 3.9 | C_19_H_28_O_11_ | 1′-O-β-D-(4-hydroxyphenethyl)-β-D-apigenose- (1 → 2) -glucoside | |
| 16 | 6.6 | C_34_H_44_O_20_ | 3,4-dihydroxyphenylethanol 8-O-β-D-Allanyl (1 → 3) - [β-D-glucosyl (1 → 6)] - 4-O-caffeoyl-β-D-glucoside | |
| 23 | 9.6 | C_27_H_30_O_15_ | 5,7,4′-trihydroxy-6-methoxy-8-C- [β-D-xylose- (1 → 2)]-β-D-glucoflavone carboside | |
| 24 | 10.2 | C_28_H_32_O_16_ | 5,3′,4′-trihydroxy-7,8-dimethoxy-6-C- [β-D-xylose- (1 → 2)]-β-D-glucoflavone carboside | |
| 25 | 10.9 | C_28_H_36_O_15_ | Nuomioside A | |
| 26 | 11.5 | C_28_H_32_O_15_ | 5,4′-dihydroxy-6,7-dimethoxy-8-C- [β-D-xylose- (1 → 2)]-β-D-glucoflavone carboside | |
| 27 | 11.9 | C_28_H_34_O_15_ | 5,3 ′, 4′-trihydroxy-6,7-dimethoxy-8-C- [β-D-apigenose- (1 → 2)]-β-D-glucoflavone carboside | |
| 29 | 12.3 | C_10_H_10_O_4_ | Ferulic acid | |
|  | | | |  |
|  | | | |  |
