# Supplementary material for: Extract of Corallodiscus flabellata attenuates renal fibrosis in SAMP8 mice via the Wnt/β-catenin/RAS signaling pathway
Source: BMC Complement Med Ther. 2022 Feb 28;22:52. doi: 10.1186/s12906-022-03535-y (PMC8887028; doi:10.1186/s12906-022-03535-y)
Supplement: Supplementary file 2 — Additional file 2. [file 12906_2022_3535_MOESM2_ESM.doc]

Data processing adopts IBM SPSS Stastic 26.0. During data processing, the numbers 1-5 in VAR00001 represent Con, M, CF-L, CF-M, CF-H, respectively. There was 1 death in the Con and M group, and 2 deaths in the CF-H group. Missing data in each group means that the mice have died or the deviation has been shaved.
IL-1β£º
Con	M	CF-L	CF-M	CF-H	
454.62	589.23	407.62	358.46	362.31	
408.52	789.23	409.46	473.85	439.23	
436.92	993.08	408.46	477.69	570	
415	1008.46	562.31	485.39	593.08	
481.54	1031.54	570	512.31	650.77	
517.81	1035.39	589.23	543.08	712.31	
658.31	1116.15	596.92	573.85	735.38	
748.83	1127.69	596.92	573.85	793.08	
	1231.54	666.15	620		
	1373.85	770.62	658.46		
		776.92	662.31		
			816.15		


Descriptives	
VAR00002  	
	N	Mean	Std. Deviation	Std. Error	95% Confidence Interval for Mean	Minimum	Maximum	
					Lower Bound	Upper Bound			
1	8	515.1938	123.87875	43.79775	411.6285	618.7590	408.52	748.83	
2	10	1029.6160	218.56139	69.11518	873.2666	1185.9654	589.23	1373.85	
3	11	577.6918	131.06186	39.51664	489.6433	665.7404	407.62	776.92	
4	12	562.9500	117.78106	34.00046	488.1155	637.7845	358.46	816.15	
5	8	607.0200	148.17747	52.38865	483.1405	730.8995	362.31	793.08	
Total	49	660.8955	239.76027	34.25147	592.0283	729.7627	358.46	1373.85	

Test of Homogeneity of Variances	
	Levene Statistic	df1	df2	Sig.	
VAR00002	Based on Mean	.624	4	44	.648	
	Based on Median	.624	4	44	.648	
	Based on Median and with adjusted df	.624	4	30.879	.649	
	Based on trimmed mean	.618	4	44	.652	

ANOVA	
VAR00002  	
	Sum of Squares	df	Mean Square	F	Sig.	
Between Groups	1743871.858	4	435967.964	18.892	.000	
Within Groups	1015407.559	44	23077.445			
Total	2759279.417	48				


Robust Tests of Equality of Means	
VAR00002  	
	Statistica	df1	df2	Sig.	
Welch	10.006	4	20.608	.000	
Brown-Forsythe	18.746	4	33.272	.000	
a. Asymptotically F distributed.	

Post Hoc Tests

Multiple Comparisons	
Dependent Variable:   VAR00002  	
	(I) VAR00001	(J) VAR00001	Mean Difference (I-J)	Std. Error	Sig.	95% Confidence Interval	
						Lower Bound	Upper Bound	
LSD	1	2	-514.42225*	72.05848	.000	-659.6466	-369.1979	
		3	-62.49807	70.58775	.381	-204.7583	79.7622	
		4	-47.75625	69.33831	.495	-187.4984	91.9859	
		5	-91.82625	75.95631	.233	-244.9061	61.2536	
	2	1	514.42225*	72.05848	.000	369.1979	659.6466	
		3	451.92418*	66.37540	.000	318.1533	585.6950	
		4	466.66600*	65.04510	.000	335.5762	597.7558	
		5	422.59600*	72.05848	.000	277.3717	567.8203	
	3	1	62.49807	70.58775	.381	-79.7622	204.7583	
		2	-451.92418*	66.37540	.000	-585.6950	-318.1533	
		4	14.74182	63.41191	.817	-113.0565	142.5401	
		5	-29.32818	70.58775	.680	-171.5884	112.9321	
	4	1	47.75625	69.33831	.495	-91.9859	187.4984	
		2	-466.66600*	65.04510	.000	-597.7558	-335.5762	
		3	-14.74182	63.41191	.817	-142.5401	113.0565	
		5	-44.07000	69.33831	.528	-183.8122	95.6722	
	5	1	91.82625	75.95631	.233	-61.2536	244.9061	
		2	-422.59600*	72.05848	.000	-567.8203	-277.3717	
		3	29.32818	70.58775	.680	-112.9321	171.5884	
		4	44.07000	69.33831	.528	-95.6722	183.8122	
Tamhane	1	2	-514.42225*	81.82390	.000	-783.4710	-245.3735	
		3	-62.49807	58.98990	.974	-254.1865	129.1904	
		4	-47.75625	55.44614	.994	-230.1552	134.6427	
		5	-91.82625	68.28480	.894	-319.4160	135.7635	
	2	1	514.42225*	81.82390	.000	245.3735	783.4710	
		3	451.92418*	79.61453	.001	189.5576	714.2907	
		4	466.66600*	77.02558	.000	208.7656	724.5664	
		5	422.59600*	86.72646	.002	140.5741	704.6179	
	3	1	62.49807	58.98990	.974	-129.1904	254.1865	
		2	-451.92418*	79.61453	.001	-714.2907	-189.5576	
		4	14.74182	52.13057	1.000	-148.9094	178.3931	
		5	-29.32818	65.62115	1.000	-246.6725	188.0161	
	4	1	47.75625	55.44614	.994	-134.6427	230.1552	
		2	-466.66600*	77.02558	.000	-724.5664	-208.7656	
		3	-14.74182	52.13057	1.000	-178.3931	148.9094	
		5	-44.07000	62.45480	.999	-254.9083	166.7683	
	5	1	91.82625	68.28480	.894	-135.7635	319.4160	
		2	-422.59600*	86.72646	.002	-704.6179	-140.5741	
		3	29.32818	65.62115	1.000	-188.0161	246.6725	
		4	44.07000	62.45480	.999	-166.7683	254.9083	
Dunnett t (2-sided)b	1	5	-91.82625	75.95631	.548	-282.9684	99.3159	
	2	5	422.59600*	72.05848	.000	241.2626	603.9294	
	3	5	-29.32818	70.58775	.979	-206.9605	148.3041	
	4	5	-44.07000	69.33831	.911	-218.5581	130.4181	
*. The mean difference is significant at the 0.05 level.	
b. Dunnett t-tests treat one group as a control, and compare all other groups against it.	

Homogeneous Subsets

VAR00002	
	VAR00001	N	Subset for alpha = 0.05	
			1	2	
Student-Newman-Keulsa,b	1	8	515.1938		
	4	12	562.9500		
	3	11	577.6918		
	5	8	607.0200		
	2	10		1029.6160	
	Sig.		.555	1.000	
Waller-Duncana,b,c	1	8	515.1938		
	4	12	562.9500		
	3	11	577.6918		
	5	8	607.0200		
	2	10		1029.6160	
Means for groups in homogeneous subsets are displayed.	
a. Uses Harmonic Mean Sample Size = 9.538.	
b. The group sizes are unequal. The harmonic mean of the group sizes is used. Type I error levels are not guaranteed.	
c. Type 1/Type 2 Error Seriousness Ratio = 100.	

Means Plots


TNF-α£º
Con	M	CF-L	CF-M	CF-H	
224.206	323.927	166.775	189.607	192.475	
233.322	326.976	161.099	181.022	285.616	
212.629	279.725	144.155	130.138	203.975	
198.733	245.823	105.172	169.618	201.096	
175.733	201.775	132.933	138.536	183.881	
141.776	206.775	138.536	132.933	141.344	
	205.607	149.789	141.344	172.464	
	321.91	172.464	161.099	152.611	
		189.607	141.344	135.733	
			149.789		

Oneway


Descriptives	
VAR00002  	
	N	Mean	Std. Deviation	Std. Error	95% Confidence Interval for Mean	Minimum	Maximum	
					Lower Bound	Upper Bound			
1	6	197.7332	34.07484	13.91099	161.9738	233.4925	141.78	233.32	
2	8	264.0648	56.11828	19.84081	217.1487	310.9808	201.78	326.98	
3	9	151.1700	24.82961	8.27654	132.0843	170.2557	105.17	189.61	
4	10	153.5430	20.76510	6.56650	138.6885	168.3975	130.14	189.61	
5	9	185.4661	45.19011	15.06337	150.7299	220.2023	135.73	285.62	
Total	42	187.2398	54.98777	8.48480	170.1044	204.3752	105.17	326.98	

Test of Homogeneity of Variances	
	Levene Statistic	df1	df2	Sig.	
VAR00002	Based on Mean	3.520	4	37	.016	
	Based on Median	3.281	4	37	.021	
	Based on Median and with adjusted df	3.281	4	25.898	.027	
	Based on trimmed mean	3.479	4	37	.016	

ANOVA	
VAR00002  	
	Sum of Squares	df	Mean Square	F	Sig.	
Between Groups	70969.579	4	17742.395	12.386	.000	
Within Groups	53000.253	37	1432.439			
Total	123969.832	41				

Robust Tests of Equality of Means	
VAR00002  	
	Statistica	df1	df2	Sig.	
Welch	8.492	4	16.403	.001	
Brown-Forsythe	11.903	4	23.861	.000	
a. Asymptotically F distributed.	

Post Hoc Tests


Multiple Comparisons	
Dependent Variable:   VAR00002  	
	(I) VAR00001	(J) VAR00001	Mean Difference (I-J)	Std. Error	Sig.	95% Confidence Interval	
						Lower Bound	Upper Bound	
LSD	1	2	-66.33158*	20.44003	.002	-107.7470	-24.9162	
		3	46.56317*	19.94743	.025	6.1458	86.9805	
		4	44.19017*	19.54441	.030	4.5894	83.7909	
		5	12.26706	19.94743	.542	-28.1503	52.6844	
	2	1	66.33158*	20.44003	.002	24.9162	107.7470	
		3	112.89475*	18.39062	.000	75.6318	150.1577	
		4	110.52175*	17.95268	.000	74.1462	146.8973	
		5	78.59864*	18.39062	.000	41.3357	115.8616	
	3	1	-46.56317*	19.94743	.025	-86.9805	-6.1458	
		2	-112.89475*	18.39062	.000	-150.1577	-75.6318	
		4	-2.37300	17.38976	.892	-37.6080	32.8620	
		5	-34.29611	17.84152	.062	-70.4465	1.8542	
	4	1	-44.19017*	19.54441	.030	-83.7909	-4.5894	
		2	-110.52175*	17.95268	.000	-146.8973	-74.1462	
		3	2.37300	17.38976	.892	-32.8620	37.6080	
		5	-31.92311	17.38976	.074	-67.1581	3.3119	
	5	1	-12.26706	19.94743	.542	-52.6844	28.1503	
		2	-78.59864*	18.39062	.000	-115.8616	-41.3357	
		3	34.29611	17.84152	.062	-1.8542	70.4465	
		4	31.92311	17.38976	.074	-3.3119	67.1581	
Tamhane	1	2	-66.33158	24.23166	.170	-149.6631	16.9999	
		3	46.56317	16.18693	.178	-13.9807	107.1071	
		4	44.19017	15.38294	.207	-16.5809	104.9612	
		5	12.26706	20.50417	1.000	-56.9647	81.4988	
	2	1	66.33158	24.23166	.170	-16.9999	149.6631	
		3	112.89475*	21.49788	.005	34.8859	190.9036	
		4	110.52175*	20.89920	.006	32.4613	188.5822	
		5	78.59864	24.91110	.071	-4.5394	161.7367	
	3	1	-46.56317	16.18693	.178	-107.1071	13.9807	
		2	-112.89475*	21.49788	.005	-190.9036	-34.8859	
		4	-2.37300	10.56504	1.000	-36.7137	31.9677	
		5	-34.29611	17.18738	.508	-92.5792	23.9870	
	4	1	-44.19017	15.38294	.207	-104.9612	16.5809	
		2	-110.52175*	20.89920	.006	-188.5822	-32.4613	
		3	2.37300	10.56504	1.000	-31.9677	36.7137	
		5	-31.92311	16.43241	.557	-89.1949	25.3486	
	5	1	-12.26706	20.50417	1.000	-81.4988	56.9647	
		2	-78.59864	24.91110	.071	-161.7367	4.5394	
		3	34.29611	17.18738	.508	-23.9870	92.5792	
		4	31.92311	16.43241	.557	-25.3486	89.1949	
Dunnett t (2-sided)b	1	5	12.26706	19.94743	.931	-38.7609	63.2950	
	2	5	78.59864*	18.39062	.000	31.5532	125.6441	
	3	5	-34.29611	17.84152	.190	-79.9369	11.3447	
	4	5	-31.92311	17.38976	.222	-76.4083	12.5620	
*. The mean difference is significant at the 0.05 level.	
b. Dunnett t-tests treat one group as a control, and compare all other groups against it.	

Homogeneous Subsets

VAR00002	
	VAR00001	N	Subset for alpha = 0.05	
			1	2	
Student-Newman-Keulsa,b	3	9	151.1700		
	4	10	153.5430		
	5	9	185.4661		
	1	6	197.7332		
	2	8		264.0648	
	Sig.		.079	1.000	
Waller-Duncana,b,c	3	9	151.1700		
	4	10	153.5430		
	5	9	185.4661		
	1	6	197.7332		
	2	8		264.0648	
Means for groups in homogeneous subsets are displayed.	
a. Uses Harmonic Mean Sample Size = 8.145.	
b. The group sizes are unequal. The harmonic mean of the group sizes is used. Type I error levels are not guaranteed.	
c. Type 1/Type 2 Error Seriousness Ratio = 100.	

Means Plots


Serum urea nitrogen:
Con	M	CF-L	CF-M	CF-H	
452.601	645.0549	793.9927	389.3773	630.4029	
487.653	661.1722	889.9275	451.6484	762.2711	
458.974	835.5311	858.7679	459.707	820.1465	
546.586	1032.601	855.5788	525.641	887.5458	
645.055	1056.777	860.4896	721.978	938.8278	
867.739	1086.813	1045.855	853.1136	974.7253	
	1102.93	1077.289	938.0952	1001.099	
	1144.689	1110.63	1044.322	1025.275	
	1207.692			365.2015	
	1262.637			1206.227	

Oneway

Descriptives	
VAR00002  	
	N	Mean	Std. Deviation	Std. Error	95% Confidence Interval for Mean	Minimum	Maximum	
					Lower Bound	Upper Bound			
1	6	576.4347	159.66958	65.18483	408.8717	743.9976	452.60	867.74	
2	10	1003.5897	216.84052	68.57099	848.4714	1158.7081	645.05	1262.64	
3	8	936.5664	121.27114	42.87582	835.1811	1037.9516	793.99	1110.63	
4	8	672.9853	250.61139	88.60451	463.4690	882.5017	389.38	1044.32	
5	10	861.1722	234.73348	74.22924	693.2539	1029.0904	365.20	1206.23	
Total	42	832.9200	250.54491	38.65992	754.8447	910.9953	365.20	1262.64	


Test of Homogeneity of Variances	
	Levene Statistic	df1	df2	Sig.	
VAR00002	Based on Mean	1.316	4	37	.282	
	Based on Median	.989	4	37	.426	
	Based on Median and with adjusted df	.989	4	32.063	.428	
	Based on trimmed mean	1.278	4	37	.296	


ANOVA	
VAR00002  	
	Sum of Squares	df	Mean Square	F	Sig.	
Between Groups	984545.089	4	246136.272	5.731	.001	
Within Groups	1589137.741	37	42949.669			
Total	2573682.831	41				


Robust Tests of Equality of Means	
VAR00002  	
	Statistica	df1	df2	Sig.	
Welch	6.885	4	17.436	.002	
Brown-Forsythe	6.062	4	32.030	.001	
a. Asymptotically F distributed.	


Post Hoc Tests


Multiple Comparisons	
Dependent Variable:   VAR00002  	
	(I) VAR00001	(J) VAR00001	Mean Difference (I-J)	Std. Error	Sig.	95% Confidence Interval	
						Lower Bound	Upper Bound	
LSD	1	2	-427.15508*	107.01983	.000	-643.9979	-210.3123	
		3	-360.13168*	111.92402	.003	-586.9113	-133.3521	
		4	-96.55068	111.92402	.394	-323.3303	130.2289	
		5	-284.73749*	107.01983	.011	-501.5803	-67.8947	
	2	1	427.15508*	107.01983	.000	210.3123	643.9979	
		3	67.02339	98.30400	.500	-132.1594	266.2062	
		4	330.60440*	98.30400	.002	131.4216	529.7872	
		5	142.41758	92.68190	.133	-45.3738	330.2089	
	3	1	360.13168*	111.92402	.003	133.3521	586.9113	
		2	-67.02339	98.30400	.500	-266.2062	132.1594	
		4	263.58100*	103.62151	.015	53.6239	473.5381	
		5	75.39419	98.30400	.448	-123.7886	274.5770	
	4	1	96.55068	111.92402	.394	-130.2289	323.3303	
		2	-330.60440*	98.30400	.002	-529.7872	-131.4216	
		3	-263.58100*	103.62151	.015	-473.5381	-53.6239	
		5	-188.18681	98.30400	.063	-387.3696	10.9960	
	5	1	284.73749*	107.01983	.011	67.8947	501.5803	
		2	-142.41758	92.68190	.133	-330.2089	45.3738	
		3	-75.39419	98.30400	.448	-274.5770	123.7886	
		4	188.18681	98.30400	.063	-10.9960	387.3696	
Tamhane	1	2	-427.15508*	94.60996	.006	-744.1306	-110.1795	
		3	-360.13168*	78.02178	.012	-646.3575	-73.9059	
		4	-96.55068	109.99919	.994	-473.7325	280.6311	
		5	-284.73749	98.78787	.117	-613.7114	44.2365	
	2	1	427.15508*	94.60996	.006	110.1795	744.1306	
		3	67.02339	80.87223	.996	-199.1788	333.2256	
		4	330.60440	112.03901	.100	-40.7473	701.9561	
		5	142.41758	101.05425	.856	-179.8276	464.6628	
	3	1	360.13168*	78.02178	.012	73.9059	646.3575	
		2	-67.02339	80.87223	.996	-333.2256	199.1788	
		4	263.58100	98.43320	.207	-86.6011	613.7631	
		5	75.39419	85.72232	.993	-208.6908	359.4792	
	4	1	96.55068	109.99919	.994	-280.6311	473.7325	
		2	-330.60440	112.03901	.100	-701.9561	40.7473	
		3	-263.58100	98.43320	.207	-613.7631	86.6011	
		5	-188.18681	115.58866	.736	-568.1944	191.8208	
	5	1	284.73749	98.78787	.117	-44.2365	613.7114	
		2	-142.41758	101.05425	.856	-464.6628	179.8276	
		3	-75.39419	85.72232	.993	-359.4792	208.6908	
		4	188.18681	115.58866	.736	-191.8208	568.1944	
Dunnett t (2-sided)b	1	5	-284.73749*	107.01983	.040	-559.3533	-10.1217	
	2	5	142.41758	92.68190	.375	-95.4067	380.2418	
	3	5	75.39419	98.30400	.869	-176.8565	327.6449	
	4	5	-188.18681	98.30400	.197	-440.4375	64.0639	
*. The mean difference is significant at the 0.05 level.	
b. Dunnett t-tests treat one group as a control, and compare all other groups against it.	

Homogeneous Subsets

VAR00002	
	VAR00001	N	Subset for alpha = 0.05	
			1	2	3	
Student-Newman-Keulsa,b	1	6	576.4347			
	4	8	672.9853	672.9853		
	5	10		861.1722	861.1722	
	3	8			936.5664	
	2	10			1003.5897	
	Sig.		.354	.076	.360	
Waller-Duncana,b,c	1	6	576.4347			
	4	8	672.9853	672.9853		
	5	10		861.1722	861.1722	
	3	8			936.5664	
	2	10			1003.5897	
Means for groups in homogeneous subsets are displayed.	
a. Uses Harmonic Mean Sample Size = 8.108.	
b. The group sizes are unequal. The harmonic mean of the group sizes is used. Type I error levels are not guaranteed.	
c. Type 1/Type 2 Error Seriousness Ratio = 100.	


Means Plots


serum Cr:
Con	M	CF-L	CF-M	CF-H	
718.14	1172.68	876.45	709.68	1654.14	
1343.67	1382.51	754.1	906.25	835.9	
1338.92	1188.28	1060.92	1389.09	1086.42	
1216.57	1644.35	1243.68	1346.72	1040.79	
1172.22	1575.37	1373.47	1395.21	1649.57	
812.19	1802.79	1541.62	1382.61	1560.36	
	1890.12				
	1865.5				


Oneway

Descriptives	
VAR00002  	
	N	Mean	Std. Deviation	Std. Error	95% Confidence Interval for Mean	Minimum	Maximum	
					Lower Bound	Upper Bound			
1	6	1100.2850	269.78262	110.13829	817.1655	1383.4045	718.14	1343.67	
2	8	1565.2000	289.84886	102.47705	1322.8803	1807.5197	1172.68	1890.12	
3	6	1141.7067	300.38974	122.63360	826.4670	1456.9464	754.10	1541.62	
4	6	1188.2600	301.53306	123.10036	871.8205	1504.6995	709.68	1395.21	
5	6	1304.5300	358.74046	146.45518	928.0550	1681.0050	835.90	1654.14	
Total	32	1279.0716	336.70998	59.52248	1157.6747	1400.4685	709.68	1890.12	

Test of Homogeneity of Variances	
	Levene Statistic	df1	df2	Sig.	
VAR00002	Based on Mean	.536	4	27	.711	
	Based on Median	.423	4	27	.791	
	Based on Median and with adjusted df	.423	4	17.358	.790	
	Based on trimmed mean	.522	4	27	.720	

ANOVA	
VAR00002  	
	Sum of Squares	df	Mean Square	F	Sig.	
Between Groups	1013327.591	4	253331.898	2.735	.050	
Within Groups	2501254.337	27	92639.050			
Total	3514581.928	31				

Robust Tests of Equality of Means	
VAR00002  	
	Statistica	df1	df2	Sig.	
Welch	2.657	4	13.084	.080	
Brown-Forsythe	2.710	4	24.876	.053	
a. Asymptotically F distributed.	


Post Hoc Tests


Multiple Comparisons	
Dependent Variable:   VAR00002  	
	(I) VAR00001	(J) VAR00001	Mean Difference (I-J)	Std. Error	Sig.	95% Confidence Interval	
						Lower Bound	Upper Bound	
LSD	1	2	-464.91500*	164.37677	.009	-802.1883	-127.6417	
		3	-41.42167	175.72616	.815	-401.9820	319.1386	
		4	-87.97500	175.72616	.621	-448.5353	272.5853	
		5	-204.24500	175.72616	.255	-564.8053	156.3153	
	2	1	464.91500*	164.37677	.009	127.6417	802.1883	
		3	423.49333*	164.37677	.016	86.2201	760.7666	
		4	376.94000*	164.37677	.030	39.6667	714.2133	
		5	260.67000	164.37677	.124	-76.6033	597.9433	
	3	1	41.42167	175.72616	.815	-319.1386	401.9820	
		2	-423.49333*	164.37677	.016	-760.7666	-86.2201	
		4	-46.55333	175.72616	.793	-407.1136	314.0070	
		5	-162.82333	175.72616	.362	-523.3836	197.7370	
	4	1	87.97500	175.72616	.621	-272.5853	448.5353	
		2	-376.94000*	164.37677	.030	-714.2133	-39.6667	
		3	46.55333	175.72616	.793	-314.0070	407.1136	
		5	-116.27000	175.72616	.514	-476.8303	244.2903	
	5	1	204.24500	175.72616	.255	-156.3153	564.8053	
		2	-260.67000	164.37677	.124	-597.9433	76.6033	
		3	162.82333	175.72616	.362	-197.7370	523.3836	
		4	116.27000	175.72616	.514	-244.2903	476.8303	
Tamhane	1	2	-464.91500	150.43932	.095	-985.3276	55.4976	
		3	-41.42167	164.83156	1.000	-631.2581	548.4148	
		4	-87.97500	165.17912	1.000	-679.1831	503.2331	
		5	-204.24500	183.24727	.969	-871.5189	463.0289	
	2	1	464.91500	150.43932	.095	-55.4976	985.3276	
		3	423.49333	159.81409	.208	-136.9971	983.9838	
		4	376.94000	160.17254	.328	-185.1280	939.0080	
		5	260.67000	178.74749	.858	-386.5193	907.8593	
	3	1	41.42167	164.83156	1.000	-548.4148	631.2581	
		2	-423.49333	159.81409	.208	-983.9838	136.9971	
		4	-46.55333	173.76046	1.000	-666.4622	573.3555	
		5	-162.82333	191.01864	.995	-849.8928	524.2461	
	4	1	87.97500	165.17912	1.000	-503.2331	679.1831	
		2	-376.94000	160.17254	.328	-939.0080	185.1280	
		3	46.55333	173.76046	1.000	-573.3555	666.4622	
		5	-116.27000	191.31863	1.000	-804.1845	571.6445	
	5	1	204.24500	183.24727	.969	-463.0289	871.5189	
		2	-260.67000	178.74749	.858	-907.8593	386.5193	
		3	162.82333	191.01864	.995	-524.2461	849.8928	
		4	116.27000	191.31863	1.000	-571.6445	804.1845	
Dunnett t (2-sided)b	1	5	-204.24500	175.72616	.597	-659.1690	250.6790	
	2	5	260.67000	164.37677	.335	-164.8724	686.2124	
	3	5	-162.82333	175.72616	.757	-617.7473	292.1007	
	4	5	-116.27000	175.72616	.906	-571.1940	338.6540	
*. The mean difference is significant at the 0.05 level.	
b. Dunnett t-tests treat one group as a control, and compare all other groups against it.	

Homogeneous Subsets

VAR00002	
	VAR00001	N	Subset for alpha = 0.05	
			1	2	
Student-Newman-Keulsa,b	1	6	1100.2850		
	3	6	1141.7067		
	4	6	1188.2600		
	5	6	1304.5300		
	2	8	1565.2000		
	Sig.		.078		
Waller-Duncana,b,c	1	6	1100.2850		
	3	6	1141.7067	1141.7067	
	4	6	1188.2600	1188.2600	
	5	6	1304.5300	1304.5300	
	2	8		1565.2000	
Means for groups in homogeneous subsets are displayed.	
a. Uses Harmonic Mean Sample Size = 6.316.	
b. The group sizes are unequal. The harmonic mean of the group sizes is used. Type I error levels are not guaranteed.	
c. Type 1/Type 2 Error Seriousness Ratio = 100.	

Means Plots


urine protein:
Con	M	CF-L	CF-M	CF-H	
10.143	21.015	21.015	15.591	9.383	
12.199	14.015	17.055	15.681	11.423	
10.607	24.178	13.663	11.343	13.055	
13.871	24.178	17.451	8.101	13.463	
15.135	27.634	20.399	8.421	13.463	
15.559	28.966	23.662	13.055	15.503	
	29.982	24.278	13.541	15.911	
	30.753	20.886	16.025	17.135	
		19.874	13.754		
			13.002		

Oneway

Descriptives	
VAR00002  	
	N	Mean	Std. Deviation	Std. Error	95% Confidence Interval for Mean	Minimum	Maximum	
					Lower Bound	Upper Bound			
1	6	12.91900	2.295930	.937310	10.50957	15.32843	10.143	15.559	
2	8	25.09013	5.585968	1.974938	20.42014	29.76011	14.015	30.753	
3	9	19.80922	3.329832	1.109944	17.24969	22.36876	13.663	24.278	
4	10	12.85140	2.816778	.890744	10.83640	14.86640	8.101	16.025	
5	8	13.66700	2.515081	.889215	11.56434	15.76966	9.383	17.135	
Total	41	16.93580	5.925649	.925431	15.06544	18.80617	8.101	30.753	

Test of Homogeneity of Variances	
	Levene Statistic	df1	df2	Sig.	
VAR00002	Based on Mean	1.802	4	36	.150	
	Based on Median	1.668	4	36	.179	
	Based on Median and with adjusted df	1.668	4	23.397	.191	
	Based on trimmed mean	1.777	4	36	.155	

ANOVA	
VAR00002  	
	Sum of Squares	df	Mean Square	F	Sig.	
Between Groups	955.365	4	238.841	19.143	.000	
Within Groups	449.168	36	12.477			
Total	1404.532	40				

Robust Tests of Equality of Means	
VAR00002  	
	Statistica	df1	df2	Sig.	
Welch	12.956	4	17.189	.000	
Brown-Forsythe	19.356	4	21.905	.000	
a. Asymptotically F distributed.	

Post Hoc Tests


Multiple Comparisons	
Dependent Variable:   VAR00002  	
	(I) VAR00001	(J) VAR00001	Mean Difference (I-J)	Std. Error	Sig.	95% Confidence Interval	
						Lower Bound	Upper Bound	
LSD	1	2	-12.171125*	1.907640	.000	-16.04000	-8.30225	
		3	-6.890222*	1.861666	.001	-10.66586	-3.11459	
		4	.067600	1.824052	.971	-3.63175	3.76695	
		5	-.748000	1.907640	.697	-4.61687	3.12087	
	2	1	12.171125*	1.907640	.000	8.30225	16.04000	
		3	5.280903*	1.716371	.004	1.79994	8.76186	
		4	12.238725*	1.675499	.000	8.84066	15.63679	
		5	11.423125*	1.766131	.000	7.84124	15.00501	
	3	1	6.890222*	1.861666	.001	3.11459	10.66586	
		2	-5.280903*	1.716371	.004	-8.76186	-1.79994	
		4	6.957822*	1.622963	.000	3.66630	10.24934	
		5	6.142222*	1.716371	.001	2.66126	9.62318	
	4	1	-.067600	1.824052	.971	-3.76695	3.63175	
		2	-12.238725*	1.675499	.000	-15.63679	-8.84066	
		3	-6.957822*	1.622963	.000	-10.24934	-3.66630	
		5	-.815600	1.675499	.629	-4.21367	2.58247	
	5	1	.748000	1.907640	.697	-3.12087	4.61687	
		2	-11.423125*	1.766131	.000	-15.00501	-7.84124	
		3	-6.142222*	1.716371	.001	-9.62318	-2.66126	
		4	.815600	1.675499	.629	-2.58247	4.21367	
Tamhane	1	2	-12.171125*	2.186076	.003	-20.00986	-4.33239	
		3	-6.890222*	1.452765	.004	-11.77631	-2.00414	
		4	.067600	1.293048	1.000	-4.31461	4.44981	
		5	-.748000	1.291996	1.000	-5.20861	3.71261	
	2	1	12.171125*	2.186076	.003	4.33239	20.00986	
		3	5.280903	2.265470	.332	-2.58641	13.14822	
		4	12.238725*	2.166519	.002	4.47221	20.00524	
		5	11.423125*	2.165891	.004	3.63828	19.20797	
	3	1	6.890222*	1.452765	.004	2.00414	11.77631	
		2	-5.280903	2.265470	.332	-13.14822	2.58641	
		4	6.957822*	1.423165	.002	2.33609	11.57955	
		5	6.142222*	1.422209	.006	1.46680	10.81764	
	4	1	-.067600	1.293048	1.000	-4.44981	4.31461	
		2	-12.238725*	2.166519	.002	-20.00524	-4.47221	
		3	-6.957822*	1.423165	.002	-11.57955	-2.33609	
		5	-.815600	1.258621	.999	-4.90466	3.27346	
	5	1	.748000	1.291996	1.000	-3.71261	5.20861	
		2	-11.423125*	2.165891	.004	-19.20797	-3.63828	
		3	-6.142222*	1.422209	.006	-10.81764	-1.46680	
		4	.815600	1.258621	.999	-3.27346	4.90466	
Dunnett t (2-sided)b	1	5	-.748000	1.907640	.985	-5.61944	4.12344	
	2	5	11.423125*	1.766131	.000	6.91305	15.93320	
	3	5	6.142222*	1.716371	.004	1.75921	10.52523	
	4	5	-.815600	1.675499	.967	-5.09424	3.46304	
*. The mean difference is significant at the 0.05 level.	
b. Dunnett t-tests treat one group as a control, and compare all other groups against it.	

Homogeneous Subsets
VAR00002	
	VAR00001	N	Subset for alpha = 0.05	
			1	2	3	
Student-Newman-Keulsa,b	4	10	12.85140			
	1	6	12.91900			
	5	8	13.66700			
	3	9		19.80922		
	2	8			25.09013	
	Sig.		.890	1.000	1.000	
Waller-Duncana,b,c	4	10	12.85140			
	1	6	12.91900			
	5	8	13.66700			
	3	9		19.80922		
	2	8			25.09013	
Means for groups in homogeneous subsets are displayed.	
a. Uses Harmonic Mean Sample Size = 7.965.	
b. The group sizes are unequal. The harmonic mean of the group sizes is used. Type I error levels are not guaranteed.	
c. Type 1/Type 2 Error Seriousness Ratio = 100.	

Means Plots


12h urine volume:

Con	M	CF-L	CF-M	CF-H	
0.976	0.201	0.827	1.325	0.949	
0.89	0.242	0.667	1.255	0.6	
0.868	0.197	0.599	1.143	0.86	
0.897	0.181	0.605	1.171	0.692	
0.619	0.303	0.711	0.842	1.018	
0.489	0.313	1.124	1.284	0.914	
0.784	0.318	1.174	0.627	0.527	
0.432	0.332	0.698	1.5	0.938	
	0.183	0.755	1.603	0.514	
		0.923		0.604	

Oneway


Descriptives	
VAR00002  	
	N	Mean	Std. Deviation	Std. Error	95% Confidence Interval for Mean	Minimum	Maximum	
					Lower Bound	Upper Bound			
1	8	.74438	.204771	.072397	.57318	.91557	.432	.976	
2	9	.25222	.063841	.021280	.20315	.30129	.181	.332	
3	10	.80830	.204528	.064677	.66199	.95461	.599	1.174	
4	9	1.19444	.303813	.101271	.96091	1.42798	.627	1.603	
5	10	.76160	.193513	.061194	.62317	.90003	.514	1.018	
Total	46	.75378	.359654	.053028	.64698	.86059	.181	1.603	

Test of Homogeneity of Variances	
	Levene Statistic	df1	df2	Sig.	
VAR00002	Based on Mean	2.710	4	41	.043	
	Based on Median	1.731	4	41	.162	
	Based on Median and with adjusted df	1.731	4	23.188	.177	
	Based on trimmed mean	2.605	4	41	.050	

ANOVA	
VAR00002  	
	Sum of Squares	df	Mean Square	F	Sig.	
Between Groups	4.043	4	1.011	23.305	.000	
Within Groups	1.778	41	.043			
Total	5.821	45				

Robust Tests of Equality of Means	
VAR00002  	
	Statistica	df1	df2	Sig.	
Welch	46.676	4	18.290	.000	
Brown-Forsythe	23.207	4	28.197	.000	
a. Asymptotically F distributed.	

Post Hoc Tests

Multiple Comparisons	
Dependent Variable:   VAR00002  	
	(I) VAR00001	(J) VAR00001	Mean Difference (I-J)	Std. Error	Sig.	95% Confidence Interval	
						Lower Bound	Upper Bound	
LSD	1	2	.492153*	.101190	.000	.28779	.69651	
		3	-.063925	.098781	.521	-.26342	.13557	
		4	-.450069*	.101190	.000	-.65443	-.24571	
		5	-.017225	.098781	.862	-.21672	.18227	
	2	1	-.492153*	.101190	.000	-.69651	-.28779	
		3	-.556078*	.095683	.000	-.74931	-.36284	
		4	-.942222*	.098169	.000	-1.14048	-.74397	
		5	-.509378*	.095683	.000	-.70261	-.31614	
	3	1	.063925	.098781	.521	-.13557	.26342	
		2	.556078*	.095683	.000	.36284	.74931	
		4	-.386144*	.095683	.000	-.57938	-.19291	
		5	.046700	.093131	.619	-.14138	.23478	
	4	1	.450069*	.101190	.000	.24571	.65443	
		2	.942222*	.098169	.000	.74397	1.14048	
		3	.386144*	.095683	.000	.19291	.57938	
		5	.432844*	.095683	.000	.23961	.62608	
	5	1	.017225	.098781	.862	-.18227	.21672	
		2	.509378*	.095683	.000	.31614	.70261	
		3	-.046700	.093131	.619	-.23478	.14138	
		4	-.432844*	.095683	.000	-.62608	-.23961	
Tamhane	1	2	.492153*	.075460	.002	.20666	.77764	
		3	-.063925	.097080	.999	-.38138	.25353	
		4	-.450069*	.124488	.028	-.86229	-.03785	
		5	-.017225	.094795	1.000	-.32862	.29417	
	2	1	-.492153*	.075460	.002	-.77764	-.20666	
		3	-.556078*	.068088	.000	-.79373	-.31842	
		4	-.942222*	.103483	.000	-1.32639	-.55806	
		5	-.509378*	.064789	.000	-.73447	-.28428	
	3	1	.063925	.097080	.999	-.25353	.38138	
		2	.556078*	.068088	.000	.31842	.79373	
		4	-.386144	.120162	.062	-.78535	.01306	
		5	.046700	.089039	1.000	-.23711	.33051	
	4	1	.450069*	.124488	.028	.03785	.86229	
		2	.942222*	.103483	.000	.55806	1.32639	
		3	.386144	.120162	.062	-.01306	.78535	
		5	.432844*	.118324	.027	.03713	.82856	
	5	1	.017225	.094795	1.000	-.29417	.32862	
		2	.509378*	.064789	.000	.28428	.73447	
		3	-.046700	.089039	1.000	-.33051	.23711	
		4	-.432844*	.118324	.027	-.82856	-.03713	
Dunnett t (2-sided)b	1	5	-.017225	.098781	.999	-.26884	.23439	
	2	5	-.509378*	.095683	.000	-.75311	-.26565	
	3	5	.046700	.093131	.965	-.19053	.28393	
	4	5	.432844*	.095683	.000	.18912	.67657	
*. The mean difference is significant at the 0.05 level.	
b. Dunnett t-tests treat one group as a control, and compare all other groups against it.	

Homogeneous Subsets

VAR00002	
	VAR00001	N	Subset for alpha = 0.05	
			1	2	3	
Student-Newman-Keulsa,b	2	9	.25222			
	1	8		.74438		
	5	10		.76160		
	3	10		.80830		
	4	9			1.19444	
	Sig.		1.000	.790	1.000	
Waller-Duncana,b,c	2	9	.25222			
	1	8		.74438		
	5	10		.76160		
	3	10		.80830		
	4	9			1.19444	
Means for groups in homogeneous subsets are displayed.	
a. Uses Harmonic Mean Sample Size = 9.137.	
b. The group sizes are unequal. The harmonic mean of the group sizes is used. Type I error levels are not guaranteed.	
c. Type 1/Type 2 Error Seriousness Ratio = 100.	

Means Plots


SOD:
Con	M	CF-L	CF-M	CF-H	
1492.837	615.192	1403.385	1581.14	713.622	
1378.165	555.721	1108.907	1192.706	641.841	
1499.537	714.378	1318.574	1594.77	530.319	
1566.803	724.46	902.522	1006.544	625.667	
851.104	650.165	1432.353	900.689	1035.128	
1159.231	508.884	1000.157	1565.05	1038.234	
1395.344	641.548	808.592	1301.404	686.212	
1677.793	758.579	827.333	1262.126	826.212	
	912.858	1635.021	1415.714		
		1590.421			

Oneway

Descriptives	
VAR00002  	
	N	Mean	Std. Deviation	Std. Error	95% Confidence Interval for Mean	Minimum	Maximum	
					Lower Bound	Upper Bound			
1	8	1377.60175	261.659549	92.510621	1158.84889	1596.35461	851.104	1677.793	
2	9	675.75389	119.733296	39.911099	583.71873	767.78905	508.884	912.858	
3	10	1202.72650	312.662383	98.872527	979.06130	1426.39170	808.592	1635.021	
4	9	1313.34922	251.614929	83.871643	1119.94087	1506.75758	900.689	1594.770	
5	8	762.15437	188.911290	66.790227	604.22058	920.08817	530.319	1038.234	
Total	44	1069.25550	368.943869	55.620381	957.08631	1181.42469	508.884	1677.793	

Test of Homogeneity of Variances	
	Levene Statistic	df1	df2	Sig.	
VAR00002	Based on Mean	2.822	4	39	.038	
	Based on Median	2.467	4	39	.061	
	Based on Median and with adjusted df	2.467	4	30.874	.066	
	Based on trimmed mean	2.789	4	39	.040	

ANOVA	
VAR00002  	
	Sum of Squares	df	Mean Square	F	Sig.	
Between Groups	3623080.534	4	905770.133	15.840	.000	
Within Groups	2230061.336	39	57181.060			
Total	5853141.870	43				

Robust Tests of Equality of Means	
VAR00002  	
	Statistica	df1	df2	Sig.	
Welch	21.570	4	18.548	.000	
Brown-Forsythe	16.282	4	32.207	.000	
a. Asymptotically F distributed.	

Post Hoc Tests

Multiple Comparisons	
Dependent Variable:   VAR00002  	
	(I) VAR00001	(J) VAR00001	Mean Difference (I-J)	Std. Error	Sig.	95% Confidence Interval	
						Lower Bound	Upper Bound	
LSD	1	2	701.847861*	116.194163	.000	466.82298	936.87274	
		3	174.875250	113.427239	.131	-54.55300	404.30350	
		4	64.252528	116.194163	.583	-170.77235	299.27741	
		5	615.447375*	119.562808	.000	373.60877	857.28598	
	2	1	-701.847861*	116.194163	.000	-936.87274	-466.82298	
		3	-526.972611*	109.870638	.000	-749.20695	-304.73827	
		4	-637.595333*	112.724896	.000	-865.60296	-409.58771	
		5	-86.400486	116.194163	.462	-321.42537	148.62439	
	3	1	-174.875250	113.427239	.131	-404.30350	54.55300	
		2	526.972611*	109.870638	.000	304.73827	749.20695	
		4	-110.622722	109.870638	.320	-332.85706	111.61162	
		5	440.572125*	113.427239	.000	211.14388	670.00037	
	4	1	-64.252528	116.194163	.583	-299.27741	170.77235	
		2	637.595333*	112.724896	.000	409.58771	865.60296	
		3	110.622722	109.870638	.320	-111.61162	332.85706	
		5	551.194847*	116.194163	.000	316.16997	786.21973	
	5	1	-615.447375*	119.562808	.000	-857.28598	-373.60877	
		2	86.400486	116.194163	.462	-148.62439	321.42537	
		3	-440.572125*	113.427239	.000	-670.00037	-211.14388	
		4	-551.194847*	116.194163	.000	-786.21973	-316.16997	
Tamhane	1	2	701.847861*	100.752721	.000	337.97372	1065.72200	
		3	174.875250	135.403071	.911	-264.21328	613.96378	
		4	64.252528	124.870603	1.000	-346.53178	475.03684	
		5	615.447375*	114.101487	.001	230.46288	1000.43187	
	2	1	-701.847861*	100.752721	.000	-1065.72200	-337.97372	
		3	-526.972611*	106.623977	.004	-892.39836	-161.54686	
		4	-637.595333*	92.883520	.000	-958.19936	-316.99130	
		5	-86.400486	77.806363	.967	-354.17310	181.37213	
	3	1	-174.875250	135.403071	.911	-613.96378	264.21328	
		2	526.972611*	106.623977	.004	161.54686	892.39836	
		4	-110.622722	129.654268	.994	-527.70217	306.45673	
		5	440.572125*	119.317689	.021	50.07200	831.07225	
	4	1	-64.252528	124.870603	1.000	-475.03684	346.53178	
		2	637.595333*	92.883520	.000	316.99130	958.19936	
		3	110.622722	129.654268	.994	-306.45673	527.70217	
		5	551.194847*	107.216542	.001	198.62329	903.76640	
	5	1	-615.447375*	114.101487	.001	-1000.43187	-230.46288	
		2	86.400486	77.806363	.967	-181.37213	354.17310	
		3	-440.572125*	119.317689	.021	-831.07225	-50.07200	
		4	-551.194847*	107.216542	.001	-903.76640	-198.62329	
Dunnett t (2-sided)b	1	5	615.447375*	119.562808	.000	312.04990	918.84485	
	2	5	-86.400486	116.194163	.864	-381.24983	208.44886	
	3	5	440.572125*	113.427239	.001	152.74401	728.40024	
	4	5	551.194847*	116.194163	.000	256.34550	846.04419	
*. The mean difference is significant at the 0.05 level.	
b. Dunnett t-tests treat one group as a control, and compare all other groups against it.	

Homogeneous Subsets

VAR00002	
	VAR00001	N	Subset for alpha = 0.05	
			1	2	
Student-Newman-Keulsa,b	2	9	675.75389		
	5	8	762.15437		
	3	10		1202.72650	
	4	9		1313.34922	
	1	8		1377.60175	
	Sig.		.455	.289	
Waller-Duncana,b,c	2	9	675.75389		
	5	8	762.15437		
	3	10		1202.72650	
	4	9		1313.34922	
	1	8		1377.60175	
Means for groups in homogeneous subsets are displayed.	
a. Uses Harmonic Mean Sample Size = 8.738.	
b. The group sizes are unequal. The harmonic mean of the group sizes is used. Type I error levels are not guaranteed.	
c. Type 1/Type 2 Error Seriousness Ratio = 100.	

Means Plots


GSH-Px:
Con	M	CF-L	CF-M	CF-H	
1840	533.333	1333.333	933.333	933.333	
1200	533.333	1333.333	833.333	1066.667	
1800	666.667	933.333	1433.333	933.333	
1733.333	666.667	1066.667	1133.333	666.667	
1066.667	800	1200	866.666	533.333	
1193	800		866.666	666.667	
1433.667			1333.333		
			1533.333		

Oneway

Descriptives	
VAR00002  	
	N	Mean	Std. Deviation	Std. Error	95% Confidence Interval for Mean	Minimum	Maximum	
					Lower Bound	Upper Bound			
1	7	1466.66671	323.700759	122.347387	1167.29344	1766.03999	1066.667	1840.000	
2	6	666.66667	119.257108	48.686510	541.51401	791.81933	533.333	800.000	
3	5	1173.33320	173.845308	77.745985	957.47574	1389.19066	933.333	1333.333	
4	8	1116.66625	282.842797	100.000030	880.20375	1353.12875	833.333	1533.333	
5	6	800.00000	206.559112	84.327404	583.22951	1016.77049	533.333	1066.667	
Total	32	1058.33322	367.000038	64.877054	926.01559	1190.65084	533.333	1840.000	

Test of Homogeneity of Variances	
	Levene Statistic	df1	df2	Sig.	
VAR00002	Based on Mean	4.036	4	27	.011	
	Based on Median	2.906	4	27	.040	
	Based on Median and with adjusted df	2.906	4	20.393	.047	
	Based on trimmed mean	4.040	4	27	.011	

ANOVA	
VAR00002  	
	Sum of Squares	df	Mean Square	F	Sig.	
Between Groups	2581333.063	4	645333.266	10.931	.000	
Within Groups	1594026.809	27	59038.030			
Total	4175359.872	31				

Robust Tests of Equality of Means	
VAR00002  	
	Statistica	df1	df2	Sig.	
Welch	13.598	4	12.915	.000	
Brown-Forsythe	12.086	4	22.136	.000	
a. Asymptotically F distributed.	

Post Hoc Tests

Multiple Comparisons	
Dependent Variable:   VAR00002  	
	(I) VAR00001	(J) VAR00001	Mean Difference (I-J)	Std. Error	Sig.	95% Confidence Interval	
						Lower Bound	Upper Bound	
LSD	1	2	800.000048*	135.180161	.000	522.63327	1077.36683	
		3	293.333514*	142.273013	.049	1.41340	585.25362	
		4	350.000464*	125.752765	.010	91.97710	608.02383	
		5	666.666714*	135.180161	.000	389.29993	944.03349	
	2	1	-800.000048*	135.180161	.000	-1077.36683	-522.63327	
		3	-506.666533*	147.130138	.002	-808.55264	-204.78043	
		4	-449.999583*	131.222808	.002	-719.24655	-180.75262	
		5	-133.333333	140.283083	.350	-421.17044	154.50378	
	3	1	-293.333514*	142.273013	.049	-585.25362	-1.41340	
		2	506.666533*	147.130138	.002	204.78043	808.55264	
		4	56.666950	138.518445	.686	-227.54942	340.88332	
		5	373.333200*	147.130138	.017	71.44709	675.21931	
	4	1	-350.000464*	125.752765	.010	-608.02383	-91.97710	
		2	449.999583*	131.222808	.002	180.75262	719.24655	
		3	-56.666950	138.518445	.686	-340.88332	227.54942	
		5	316.666250*	131.222808	.023	47.41929	585.91321	
	5	1	-666.666714*	135.180161	.000	-944.03349	-389.29993	
		2	133.333333	140.283083	.350	-154.50378	421.17044	
		3	-373.333200*	147.130138	.017	-675.21931	-71.44709	
		4	-316.666250*	131.222808	.023	-585.91321	-47.41929	
Tamhane	1	2	800.000048*	131.678622	.003	293.36365	1306.63644	
		3	293.333514	144.959723	.527	-231.09080	817.75783	
		4	350.000464	158.015471	.380	-189.06229	889.06322	
		5	666.666714*	148.593385	.011	140.24321	1193.09022	
	2	1	-800.000048*	131.678622	.003	-1306.63644	-293.36365	
		3	-506.666533*	91.732298	.009	-876.72244	-136.61063	
		4	-449.999583*	111.222220	.023	-847.52798	-52.47119	
		5	-133.333333	97.372930	.903	-504.94256	238.27589	
	3	1	-293.333514	144.959723	.527	-817.75783	231.09080	
		2	506.666533*	91.732298	.009	136.61063	876.72244	
		4	56.666950	126.666666	1.000	-384.66225	497.99615	
		5	373.333200	114.697643	.095	-48.27832	794.94472	
	4	1	-350.000464	158.015471	.380	-889.06322	189.06229	
		2	449.999583*	111.222220	.023	52.47119	847.52798	
		3	-56.666950	126.666666	1.000	-497.99615	384.66225	
		5	316.666250	130.809469	.280	-130.17979	763.51229	
	5	1	-666.666714*	148.593385	.011	-1193.09022	-140.24321	
		2	133.333333	97.372930	.903	-238.27589	504.94256	
		3	-373.333200	114.697643	.095	-794.94472	48.27832	
		4	-316.666250	130.809469	.280	-763.51229	130.17979	
Dunnett t (2-sided)b	1	5	666.666714*	135.180161	.000	316.63638	1016.69705	
	2	5	-133.333333	140.283083	.742	-496.57698	229.91031	
	3	5	373.333200	147.130138	.056	-7.63995	754.30635	
	4	5	316.666250	131.222808	.073	-23.11706	656.44956	
*. The mean difference is significant at the 0.05 level.	
b. Dunnett t-tests treat one group as a control, and compare all other groups against it.	

Homogeneous Subsets

VAR00002	
	VAR00001	N	Subset for alpha = 0.05	
			1	2	3	4	
Student-Newman-Keulsa,b	2	6	666.66667				
	5	6	800.00000				
	4	8		1116.66625			
	3	5		1173.33320			
	1	7			1466.66671		
	Sig.		.341	.684	1.000		
Waller-Duncana,b,c	2	6	666.66667				
	5	6	800.00000	800.00000			
	4	8		1116.66625	1116.66625		
	3	5			1173.33320	1173.33320	
	1	7				1466.66671	
Means for groups in homogeneous subsets are displayed.	
a. Uses Harmonic Mean Sample Size = 6.241.	
b. The group sizes are unequal. The harmonic mean of the group sizes is used. Type I error levels are not guaranteed.	
c. Type 1/Type 2 Error Seriousness Ratio = 100.	

Means Plots
