# Supplementary material for: Extract of Corallodiscus flabellata attenuates renal fibrosis in SAMP8 mice via the Wnt/β-catenin/RAS signaling pathway
Source: BMC Complement Med Ther. 2022 Feb 28;22:52. doi: 10.1186/s12906-022-03535-y (PMC8887028; doi:10.1186/s12906-022-03535-y)
Supplement: Supplementary file 3 — Additional file 3. [file 12906_2022_3535_MOESM3_ESM.docx]

Panel A-I represent the whole uncropped images of the original western blots shown in **Fig3-e, 4-g** with each protein. A, p-Nrf2; B, Keap1; C, p-c-Fos; D, β-actin; E, Wnt4; F, Renin; G, AGTR1; H, GAPDH; I, β-actin. Rectangles represent image fragments placed in the manuscript. In addition, five additional figures of SA-β-gal **(Fig1a)**, Masson **(Fig1c)** and IHC **(Fig3a, 4a)** in the manuscript are also provided in sequence.

| **A.** |
| --- |
| p-Nrf2  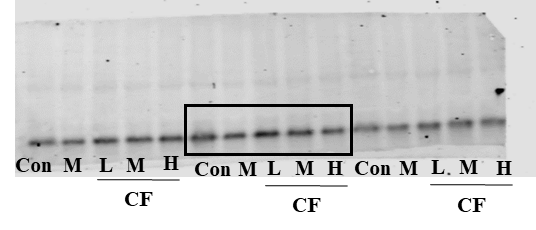  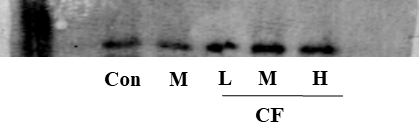  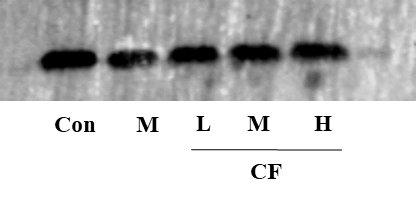 |
| **B.** |
| Keap1  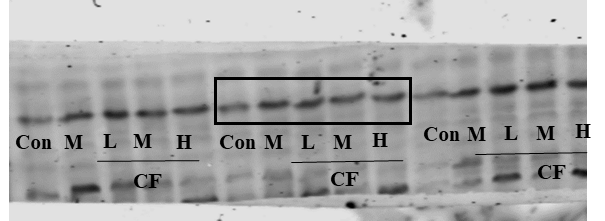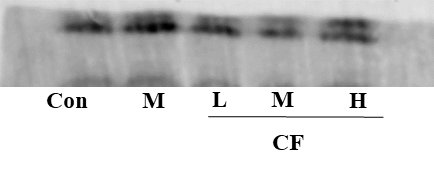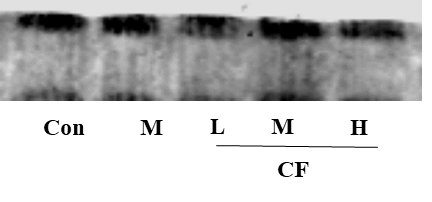 |
| **C.**  p-c-Fos  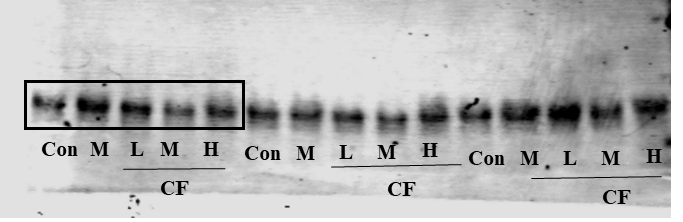 |
| 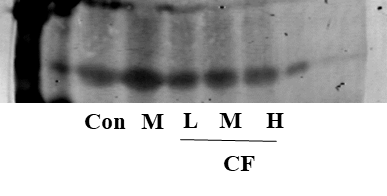  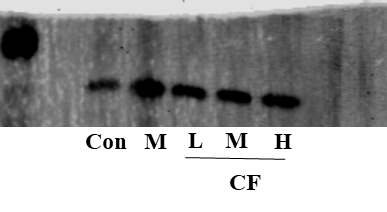 |
| **D.**  β-actin  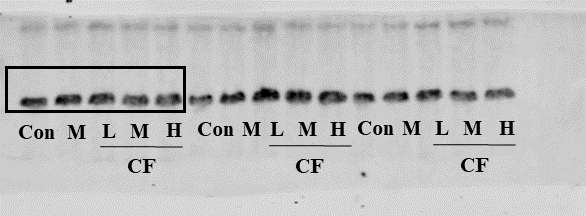  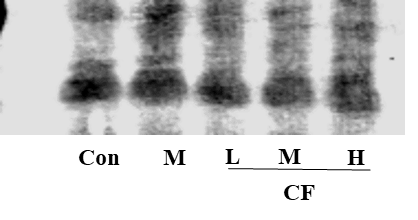 |
| 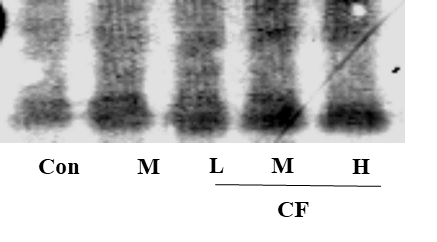 |
| **E.**  Wnt4 |
| 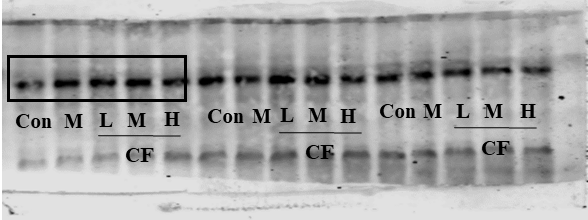  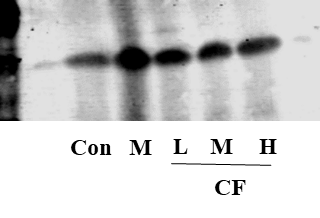  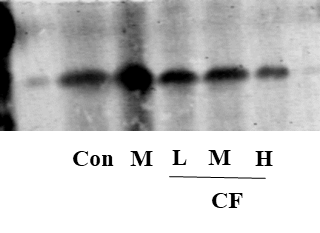 |
| **F.** Renin |
| 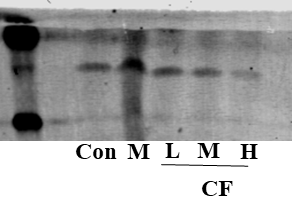  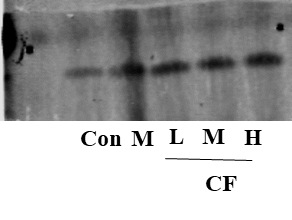  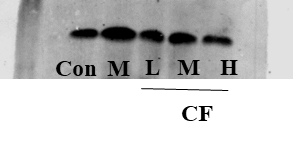 |
| **G.** AGTR1 |
| 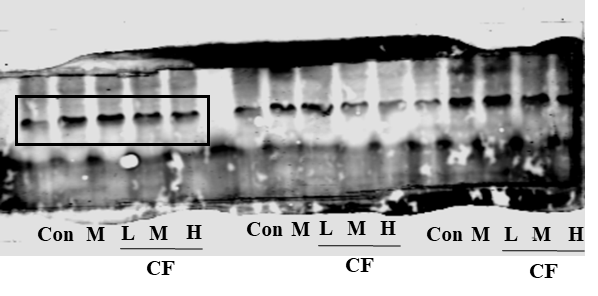  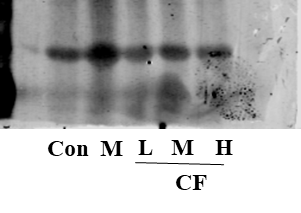  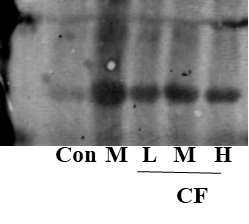 |
| **H. GAPDH** |
| 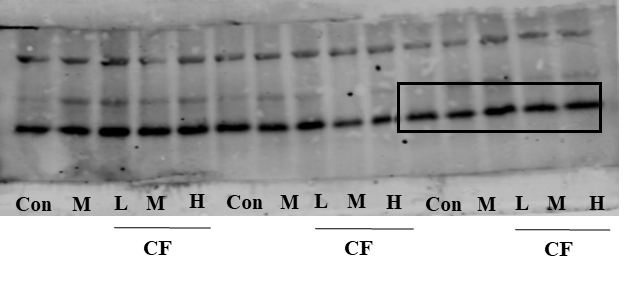  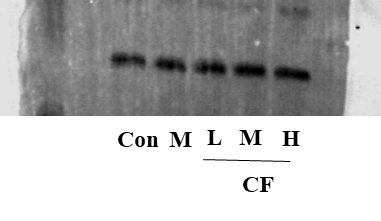  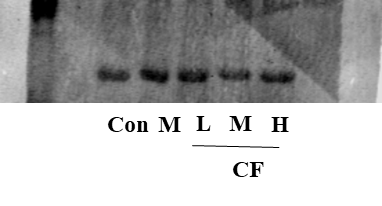 |
| **I.** β-actin  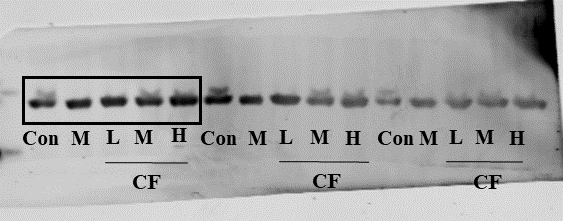 |
| 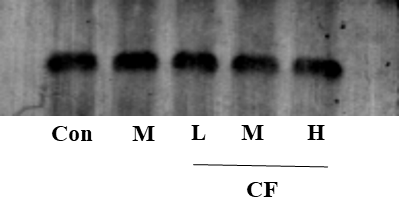  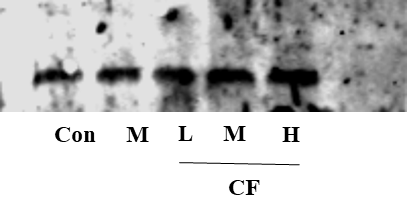 |

**SA-β-gal:**

| Con | **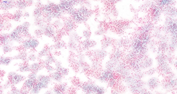** | **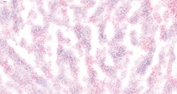** | **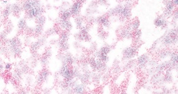** | **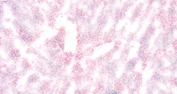** | **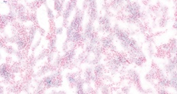** |
| --- | --- | --- | --- | --- | --- |
| M | **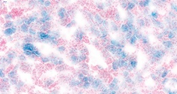** | **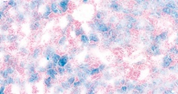** | **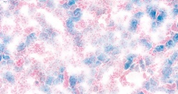** | **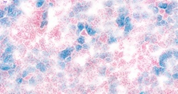** | **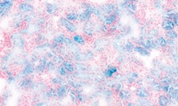** |
| CF-L | **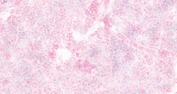** | **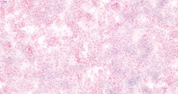** | **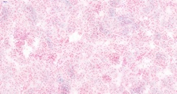** | **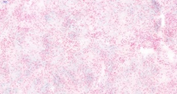** | **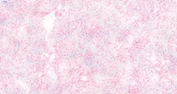** |
| CF-M | **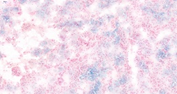** | **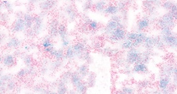** | **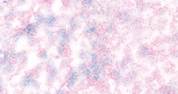** | **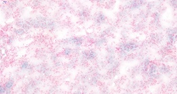** | **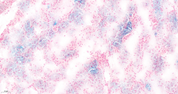** |
| CF-H | **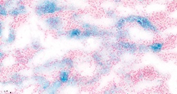** | **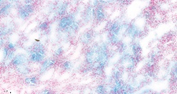** | **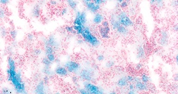** | **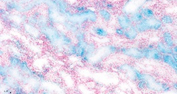** | **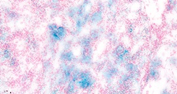** |

**Masson：**

| Con | 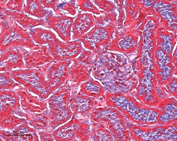 | 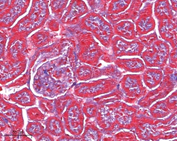 | 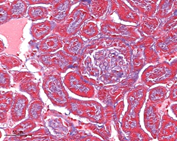 | 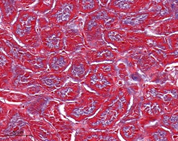 | 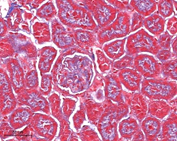 |
| --- | --- | --- | --- | --- | --- |
| M | 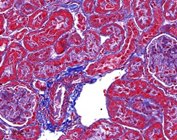 | 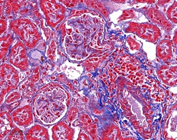 | 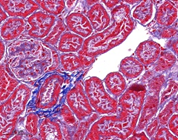 | 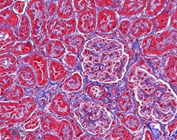 | 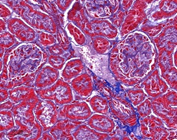 |
| CF-L | 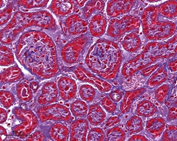 | 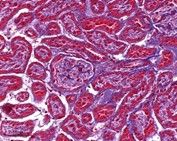 | 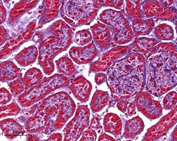 | 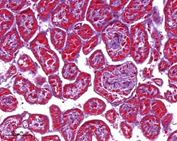 | 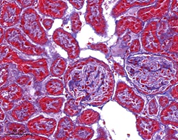 |
| CF-M | 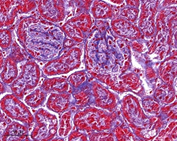 | 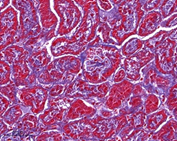 | 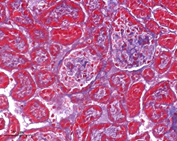 | 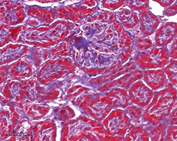 | 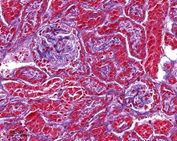 |
| CF-H | 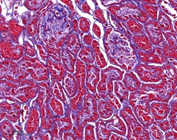 | 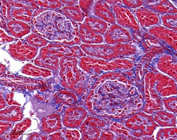 | 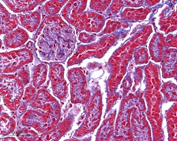 | 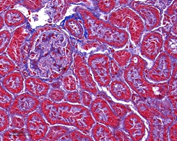 | 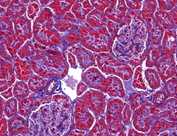 |

***p*-Nrf2:**

| Con | 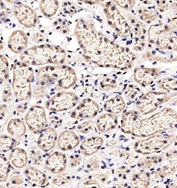 | 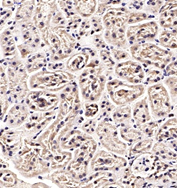 | 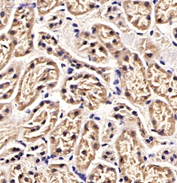 | 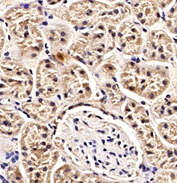 | 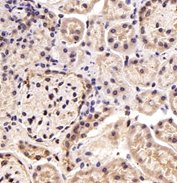 |
| --- | --- | --- | --- | --- | --- |
| M | 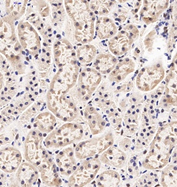 | 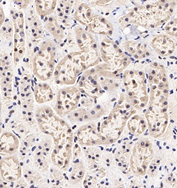 | 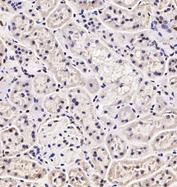 | 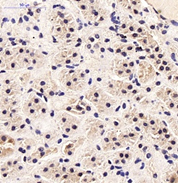 | 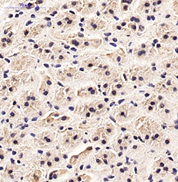 |
| CF-L | 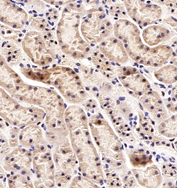 | 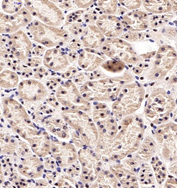 | 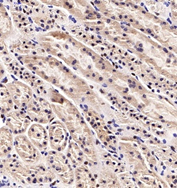 | 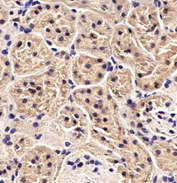 | 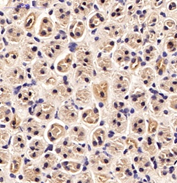 |
| CF-M | 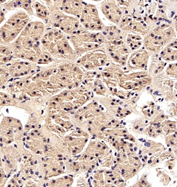 | 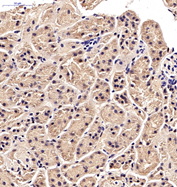 | 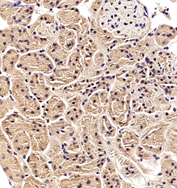 | 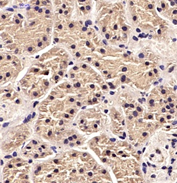 | 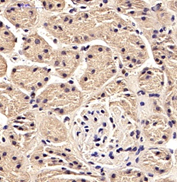 |
| CF-H | 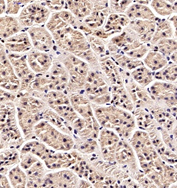 | 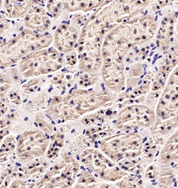 | 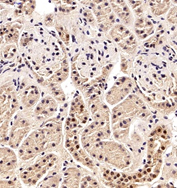 |  |  |

***p*-c-Fos**:

| Con |  |  |  |  |  |
| --- | --- | --- | --- | --- | --- |
| M |  |  |  |  |  |
| CF-L |  |  |  |  |  |
| CF-M |  |  |  |  |  |
| CF-H |  |  |  |  |  |

**AGTR1:**

| Con |  |  |  |  |  |
| --- | --- | --- | --- | --- | --- |
| M |  |  |  |  |  |
| CF-L |  |  |  |  |  |
| CF-M |  |  |  |  |  |
| CF-H |  |  |  |  |  |

**Wnt4:**

| Con |  |  |  |  |  |
| --- | --- | --- | --- | --- | --- |
| M |  |  |  |  |  |
| CF-L |  |  |  |  |  |
| CF-M |  |  |  |  |  |
| CF-H |  |  |  |  |  |

**β-catenin:**

| Con |  |  |  |  |  |
| --- | --- | --- | --- | --- | --- |
| M |  |  |  |  |  |
| CF-L |  |  |  |  |  |
| CF-M |  |  |  |  |  |
| CF-H |  |  |  |  |  |

**CTGF:**

| Con |  |  |  |  |  |
| --- | --- | --- | --- | --- | --- |
| M |  |  |  |  |  |
| CF-L |  |  |  |  |  |
| CF-M |  |  |  |  |  |
| CF-H |  |  |  |  |  |
